# The role of diffusion-weighted MRI in biological image-guided radiation therapy: a roadmap

**DOI:** 10.1088/1361-6560/ae5d80

**Published:** 2026-06-01

**Authors:** Jie Deng, Sirisha Tadimalla, Tyler M Seibert, H Michael Gach, Petra J van Houdt, David A Hormuth Ii, Xun Jia, Jiaren Zou, Christopher C Conlin, Muge Karaman, Junzhong Xu, Jill B De Vis, Lise Wei, Anna M Dornisch, Joseph Weygand, Yu-Feng Wang, Daniela Thorwarth, Xiaohong Joe Zhou, John C Gore, Xiaoyu Jiang, Taeho Kim, Annette Haworth, Heiko Enderling, Caroline Chung, Thomas E Yankeelov

**Affiliations:** 1 Department of Radiation Oncology, University of Texas Southwestern Medical Center, Dallas, TX, United States of America; 2 Department of Radiation Oncology and Molecular Radiation Sciences, Johns Hopkins University, Baltimore, MD, United States of America; 3 Department of Radiation Oncology, University of Michigan, Ann Arbor, MI, United States of America; 4 Department of Radiology, University of California San Diego Health, La Jolla, CA, United States of America; 5 Department of Radiation Medicine and Applied Sciences, University of California San Diego Health, La Jolla, CA, United States of America; 6 Department of Bioengineering, University of California San Diego Jacobs School of Engineering, La Jolla, CA, United States of America; 7 Department of Urology, University of California San Diego Health, La Jolla, CA, United States of America; 8 Institute of Medical Physics, The University of Sydney, Sydney, Australia; 9 Center for Magnetic Resonance Research, University of Illinois at Chicago, Chicago, IL, United States of America; 10 Department of Biomedical Engineering, University of Illinois at Chicago, Chicago, IL, United States of America; 11 Department of Radiology, University of Illinois at Chicago, Chicago, IL, United States of America; 12 Department of Neurosurgery, University of Illinois at Chicago, Chicago, IL, United States of America; 13 Institute of Imaging Science, Vanderbilt University Medical Center, Nashville, TN, United States of America; 14 Advanced Imaging Research Center, University of Texas Southwestern Medical Center, Dallas, TX, United States of America; 15 Department of Radiation Oncology, Washington University, St. Louis, MO, United States of America; 16 Departments of Radiology and Biomedical Engineering, Washington University, St. Louis, MO, United States of America; 17 Department of Radiation Oncology and Applied Sciences, Dartmouth College, Hanover, NH, United States of America; 18 Department of Radiation Oncology, The Netherlands Cancer Institute, Amsterdam, The Netherlands; 19 Oden Institute for Computational Engineering and Sciences, The University of Texas at Austin, Austin, Texas; 20 Departments of Biomedical Engineering, The University of Texas at Austin, Austin, TX, United States of America; 21 Diagnostic Medicine, The University of Texas at Austin, Austin, TX, United States of America; 22 Oncology, The University of Texas at Austin, Austin, TX, United States of America; 23 Livestrong Cancer Institutes, The University of Texas at Austin, Austin, TX, United States of America; 24 Department of Imaging Physics, MD Anderson Cancer Center, Houston, TX, United States of America; 25 Department of Radiation Oncology, MD Anderson Cancer Center, Houston, TX, United States of America; 26 Section for Biomedical Physics, Department of Radiation Oncology, University of Tübingen, Tübingen, Germany; 27 German Cancer Consortium, Partner site Tübingen, German Cancer Research Center, Heidelberg, Germany

**Keywords:** diffusion weighted MRI, image-guided radiation therapy, biological image-guided adaptive radiotherapy, MRI-guided radiation therapy, MR-LINAC, MRI simulator

## Abstract

This roadmap provides a comprehensive framework for integrating diffusion-weighted imaging (DWI) into radiation therapy (RT), with an emphasis on its application in magnetic resonance imaging-guided radiotherapy and its potential for driving biological image-guided adaptive radiotherapy (ART). Developed through collaboration among experts in medical physics, magnetic resonance imaging science, and radiation oncology, the paper aims to bridge disciplinary gaps and foster a shared understanding across scientific, technical, and clinical domains. It benchmarks the current state of DWI in RT, identifies critical challenges, and highlights recent advancements in acquisition, reconstruction, biophysical modeling, quality assurance, clinical validation and translation, as well as emerging concepts. By outlining ongoing efforts and forecasting future developments, this roadmap supports the adoption of DWI as a quantitative imaging biomarker for personalized and ART in precision oncology.

## Introduction—a roadmap for bridging the gap

### Jie Deng

Department of Radiation Oncology, University of Texas Southwestern Medical Center, Dallas, TX, United States of America

Magnetic resonance imaging (MRI) is increasingly being utilized to enhance image-guided RT (IGRT), playing a pivotal role in various stages of RT. These stages include creating initial dosimetry treatment plans through MR simulation, conducting mid-treatment MRI for offline replanning, and using in-treatment MRI for online adaptation of dosimetry plans to accommodate shifts in a patient’s anatomy and changes in disease state.

The recent integration of MRI with linear accelerator (LINAC) hardware to form a hybrid MR-LINAC system has allowed for MRI acquisition during each treatment session, enabling MRI-guided RT (MRIgRT). Current MRIgRT workflows optimize daily dose plans by adapting to individual patient’s anatomic changes in tumor and organs-at-risks (OARs) throughout the treatment course. However, anatomy-based adaptive radiotherapy (ART) may underestimate the true tumor extent, fail to identify tumor subregions with varying biological resistance to radiation, and delay optimal plan adaptation because that tumor size changes are downstream (weeks to months post-radiation) of *biological* changes.

To address these challenges, there is a growing focus on functional adaptation, which incorporates biological insights into the adaptive optimization process early in the radiotherapy course, known as biological image-guided ART (BIgART) (van Houdt *et al* 2021). Diffusion-weighted imaging (DWI), a well-established diagnostic technique for lesion detection, grading, and treatment response evaluation, is still in its early stages of integration into RT. Nonetheless, it has been the most extensively explored technique for incorporating biological information on MR-LINAC systems, holding promise for advancing BIgART.

**Figure 1.**
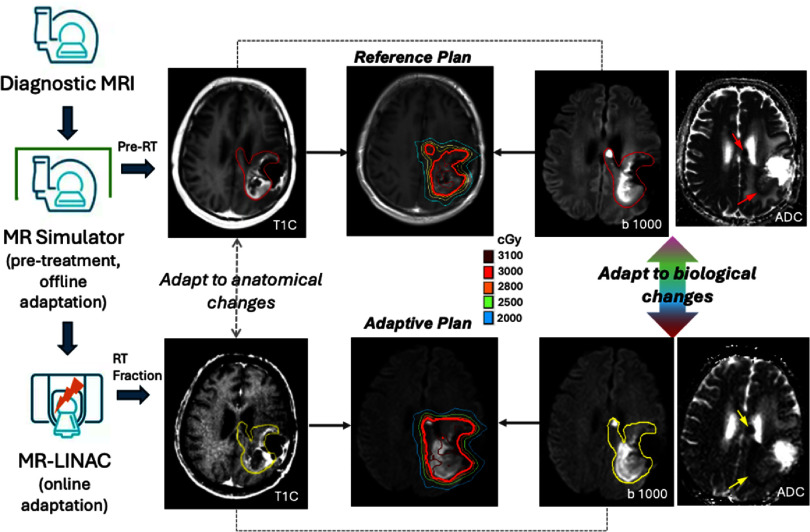
Illustration of MR-guided BIgART workflow starting anatomical adaptation and integrated with the proposed biological adaptations guided by DWI. The process begins with pre-RT MRI scans acquired during patient simulation on the MR simulator, which are used to generate a reference plan for RT. Mid-treatment images acquired on the MR simulator can be utilized for offline plan adaptation. The MR-LINAC system enables online adaptive RT by creating a daily adaptive plan that optimizes dose distribution based on anatomic variations in the tumor and OARs, delineated on anatomic MR images (e.g. *T*_1_C:*T*_1_-weighted contrast enhanced images). By incorporating biological information derived from DWI (b1000) images and calculated apparent diffusion coefficient (ADC) maps into the online re-optimization process, this workflow advances the implementation of BIgART. The colored lines on the reference plan and adaptive plan images represent radiation isodose distributions (in cGy). The single contours on the *T*_1_C and b1000 images indicate the gross tumor volume (GTV), delineated by the physician on both pre-treatment (red) and daily MR-LINAC (yellow) images. Arrows on the ADC maps highlight regions of restricted diffusion, corresponding to subregions with higher cellularity.

The MRIgRT workflow, with its current focus on anatomical adaptation and the potential for future biological adaptation guided by DWI, is illustrated in the figure 1.

This roadmap paper aims to bridge the gap between medical physicists working in both MRI and RT fields, MRI scientists and researchers, and radiation oncologists who are adopting state-of-the-art MRIgRT techniques to improve patient care. It brings together insights from experts across scientific, technical, and clinical disciplines to benchmark the current status, highlight ongoing efforts, identify existing gaps, and forecast future developments in this rapidly evolving field.

**Chapter I. DWI methods for MRIgRT from medical physicist’s perspective** provides an overview of general DWI acquisition, reconstruction, and model fitting methods for MRIgRT. A clear understanding of the hardware limitations and patient positioning configurations across different MRI platforms, particularly MRI simulators and MR-LINAC systems, is essential. This chapter discusses the options of DWI pulse sequences and protocol optimization (section 1), reconstruction methods (section 2), and biophysical model fitting (section 3). Optimizing DWI for RT requires consideration beyond the traditional tradeoffs of SNR, spatial resolution, and acquisition time, including maintaining geometric fidelity, ensuring quantitative accuracy, and accounting for biological and physiological effects. Readers will gain insight into the fundamental challenges of implementing DWI in the MRIgRT clinical setting, including acquisition strategies, image quality limitations, and the uncertainty associated with biophysical modeling.

**Chapter II. Exploring tumor microenvironment with DWI from MRI scientist’s perspective**, written by MRI scientists and researchers, presents four major categories of advanced DWI models used to probe the tumor microenvironment. Establishing the clinical relevance of DWI measurements requires linking them to underlying biological processes, such as tumor cell death, microvasculature disruption, and immune response, which occur throughout the course of radiotherapy. Readers will gain deeper understanding of the following techniques: restriction spectrum imaging (RSI), which models the diffusion signal as a linear combination of compartments and volume fractions across the diffusion spectrum (section 4); intra-voxel incoherent motion (IVIM), which characterizes microcirculation within pseudo-random vascular networks (section 5); diffusion kurtosis imaging (DKI) and fractional order calculus (FROC) models, which capture tissue heterogeneity beyond conventional diffusion metrics (section 6); MR cell size imaging, which leverages quantitative diffusion-time-dependent techniques to estimate cell size and density (section 7).

**Chapter III. Clinical perspectives on DWI applications in radiation oncology** highlights how DWI-based biomarkers can be used at different disease sites to assess their clinical utility and define its role in radiation oncology. This chapter focuses on applications in brain (section 8), head and neck (section 9), and prostate cancer (section 10). Current clinical research has primarily explored the use of DWI before RT to identify tumor subregions for dose escalation or de-escalation, predict prognosis, and evaluate treatment response after RT. However, most validation efforts have been limited to interpreting imaging data from single or a small number of time points, leaving it unclear how these findings translate to longitudinal data and whether time-series changes can inform clinical decisions regarding treatment plan adjustment. A critical open clinical question remains: Is real-time BIgART necessary for each RT fraction, enabled by treatment on MR-LINAC systems, or is intermediate, off-line adaptation to biological responses sufficient? Addressing this question will be key to defining the clinical value of integrating DWI in ART workflows.

**Chapter IV. Quality assurance/control (QA/QC), and clinical trials of DWI in RT** discusses strategies for assessing the accuracy and precision of DWI in detecting subtle treatment-related changes. Successful integration of DWI into RT workflows for informed clinical decision-making with clinical adoption requires a rigorous and standardize QA/QC approach (section 11). It also covers methods for evaluating short-term repeatability and conducting test–retest imaging studies to validate measurement robustness (section 12). Moreover, ensuring reproducibility across different imaging platforms is essential for reliable use of DWI in multi-center trials (section 13).

**Chapter V. Emerging innovations to enable adaptive RT** highlights key developments in three transformative areas: clinical implementation of DWI on MR-LINAC systems (section 14), real-time biological image-guided treatment adaptation (section 15), and anticipatory digital-twin-guided adaptation using longitudinal DWI data (section 16). Radiation oncology is rapidly evolving, driven by advances in imaging, treatment delivery, biological guidance, and mathematical tumor modeling. The availability and quantitative nature of DWI in the RT context allows for assessment of tumor biological properties across different MR platforms, positioning it as a powerful tool for realizing personalized treatment.

In this Roadmap, some degree of overlaps between contributions is inevitable. For example, recurring topics such as magnetic field inhomogeneities, imaging artifacts, variations in DWI parameter settings such as *b*-values and diffusion time, limitations of current biophysical diffusion models, reliability and interpretability of DWI measurements appear throughout the paper. These overlaps arise naturally, as each author presents the issues from different perspectives and in relation to distinct content. Given these varying emphases, it is not feasible—or desirable—to entirely isolate foundational concepts within a single section.

Major terminology and commonly used acronyms are defined in the *Major Terminology* section. Additional acronyms will be introduced in each chapter or may be repeated within individual sections of that chapter.

### Basics of DWI techniques

DWI is a quantitative MRI method to characterize tissue structure at a cellular level non-invasively (Le Bihan *et al* 1988). It applies diffusion encoding gradients designed with specific amplitude and timing to sensitize microstructural tissue water mobility (Lewis *et al* 2021, Lutsik *et al* 2024). There are three principal modes of diffusion in tissue: free, hindered, and restricted (White *et al* 2014). Free water diffusion is the random motion of water molecules in the absence of any obstacles, where the displacement distribution of the water molecules is Gaussian and characterized by the diffusion coefficient *D*. Hindered diffusion is the delay due to navigation around cellular obstacles, such as in the extracellular space/matrix. The apparent diffusion coefficient (ADC) for hindered diffusion is smaller than that of the free diffusion coefficient *D* and depends upon the tortuosity of the path that water molecules must travel around obstacles. Restricted diffusion refers to trapping of water molecules within a cell (on the time scale of DWI), such that the displacement distribution is non-Gaussian (Le Bihan 1995).

In the classical approach to DWI pioneered by Stejskal and Tanner, a pair of pulsed magnetic field gradients that are equal in magnitude but opposite in direction are applied in succession, a certain time apart, to perturb the phase of protons in the tissue (Stejskal and Tanner 1965). If there is no diffusion during the intervening period between diffusion encoding gradients, no net change in the phase of protons within the tissue would be observed, as the effect of the second pulse would reverse the effect of the first. If, however, water molecules diffuse in the time between pulses, the protons will experience a different phase offset from the second pulse than from the first, resulting in a net phase dispersion and signal attenuation (darkening) in the reconstructed image.

DWI acquisition often relies on single-shot echo planar imaging (EPI), as it captures the entire k-space after one signal excitation, resulting in very fast acquisition time and reduced sensitivity to patient motion. However, it is prone to susceptibility-induced distortions and off-resonance effects causing phase errors that accumulate during the scan, leading to image distortion and limited spatial resolution (Stehling *et al* 1991).

Diffusion models are the tool by which the DWI signal is related to quantitative information about the underlying tissue microstructure. The simplest and most widespread approach is to model the diffusion signal as if it is derived from a single compartment with Gaussian diffusion:

\begin{equation*}{ }S\left( b \right) = { }{S_0}{\mathrm{exp}}\left( { - b \cdot {\mathrm{ADC}}} \right),\end{equation*}

where *b* is the diffusion-weighting factor, or *b*-value (unit: s mm^−2^), which combines the magnitude, duration, and temporal spacing of the diffusion encoding gradients into a single parameter. For a Stejskal–Tanner diffusion gradient *G*_d_ with a duration *δ* and gradient onset separation Δ, *b*-value is defined as:

\begin{equation*}b = { }{\gamma ^2}{G_{\mathrm{d}}}^2{\delta ^2}\left( {{{\Delta }} - \delta /3} \right),\end{equation*}

and the effective diffusion time (Δ_diff_) is defined as

\begin{equation*}{{{\Delta }}_{{\mathrm{diff}}}} = {{ \Delta }} - \delta /3.\,S\left( b \right).\end{equation*}

The DWI signal ($S\left( b \right)$) is measured at a particular *b*-value, ${S_0}$ is the diffusion weighted signal without any diffusion weighting, i.e. a *b*-value of zero. ADC (unit: mm^2^ s^−1^) is the ensemble average of diffusion within all the microenvironments of the measured area (whether that is a single voxel or a larger region-of-interest) (Le Bihan *et al* 1986). It is simple to compute ADC for all voxels of an image by estimating the slope of log-transformed diffusion weighted signal as a function of *b*-value, yielding what is called an ADC map. Ideally, ADC is inversely correlated with tumor cellularity, with increased cellularity in tumors leading to more restricted diffusion, (Stejskal and Tanner 1965, Guo *et al* 2002, Chen *et al* 2005) and tumors are often identified by locally hypointense regions on an ADC map.

Major Terminology

MRI: Magnetic resonance imaging
RT: Radiation therapy/radiation treatment
IGRT: Image-guided radiotherapy
LINAC: Linear accelerator
MR-LINAC: MRI with linear accelerator
MRIgRT: Magnetic resonance imaging-guided radiotherapy
ART: Adaptive radiotherapy
BIgART: Biological image-guided adaptive radiotherapy
DWI: Diffusion weighted imaging
ADC: Apparent diffusion coefficient
*b*-value: Diffusion-weighting factor
SNR: Signal-to-noise ratio
EPI: Echo planar imaging
RF: Radiofrequency
PET: Positron emission tomography
CT: Computer tomography
AI: Artificial intelligence
QA/QC: Quality assurance/Quality control

## Chapter I. DWI methods for MRIgRT from medical physicist’s perspective

### 1. DWI acquisition on MRIgRT systems

#### Jie Deng

Department of Radiation Oncology, University of Texas Southwestern Medical Center, Dallas, TX, United States of America

##### Status

Over the past decade, significant scientific breakthroughs and engineering advancements from both the MRI and RT fields have enabled the integration of MRI with LINAC, resulting in hybrid systems that support real-time MRI guidance during RT. Currently, two major MR-LINAC systems are commercially available for clinical use: the 0.35 T MRIdian system (ViewRay Systems Inc, Ohio, USA) and the 1.5 T Unity system (Elekta AB, Stockholm, Sweden). These systems utilize horizontally oriented, closed-bore MRI hardware specifically engineered to accommodate radiation beam passage through major MRI components (cryostat, gradient coils, transmit body coil, and receive coils). However, the MRI hardware in MR-LINACs differs substantially from that in conventional diagnostic MRI scanners. These differences introduce several inherent limitations, including *B*_0_ field inhomogeneity, reduced gradient strength and slew rate, increased eddy current effects, and lower SNR (table 1). Collectively, these factors degrade overall image quality and are particularly detrimental to DWI, which relies heavily on high-performance gradients for accurate and robust acquisition (Jackson *et al* 2019, Snyder *et al* 2020, Curcuru *et al* 2021, Hunt *et al* 2022).

**Table 1.** Hardware differences between diagnostic MRI and MR-LINAC.

Parameter	Diagnostic MRI	MR-LINAC (0.35 T ViewRay MRIdian/1.5 T Elekta Unity)	Impact on image quality
	(1.5 T/3.0 T)		
Main field strength (*B*_0_)	1.5 T/3.0 T	0.35 T (MRIdian)/1.5 T (Unity)	Lower *B*_0_ leads to reduced SNR, contrast, resolution, and poorer fat water separation.
Maximum gradient Strength	45–80 mT m^−1^	<34 mT m^−1^ (Unity); ∼18 mT m^−1^ (MRIdian)	Lower gradient amplitude reduces diffusion sensitivity (max *b*-values, and effective diffusion time) and spatial resolution.
Gradient slew rate	150–200 T m s^−1^	<120 T m s^−1^ (unity); ∼200 T m s^−1^ (MRIdian)	Lower slew rates increase minimum echo time (TE), reducing SNR and increasing distortion.
Receive coil channels	16–64 channel phased-array coils	8 channels (unity); limited flex coils (MRIdian)	Fewer channels reduce parallel imaging acceleration, lower SNR, especially in deep tissue.
SNR (Normalized)	1.0 (reference)	∼0.4–0.6 (unity); ∼0.2–0.3 (MRIdian)	Lower SNR impacts the reliability of quantitative metrics such as ADC.
*B*_0_ homogeneity (within 40 cm DSV)	0.5–1 ppm	∼0.5–1.5 ppm, stable during gantry rotation (unity); ∼5 ppm, gantry-dependent (MRIdian) Gach *et al* (2020)	Poorer field homogeneity increases geometric distortion and signal dropouts, especially in DWI.
Shielding & eddy current compensation	Advanced active shielding and compensation	Limited, due to integration with LINAC	Higher eddy currents distort diffusion gradients and lead to spatial mismapping.

MRI scanners used for RT simulation (MR-simulator) are available from all major vendors and typically employ hardware similar to diagnostic MRI scanners, but with RT-specific positioning devices to ensure precise and reproducible patient setup. Although standard DWI sequences are available on MR-simulators, the integration of RT-specific components can limit the achievable image quality comparable with diagnostic MRI. Moreover, conventional DWI sequences typically rely on single-shot EPI, which is highly susceptible to geometric distortion, particularly near air-tissue interfaces in the head and neck, thorax, and abdomen. While such distortion may be acceptable in diagnostic imaging, it poses a major challenge for RT planning and guidance, where submillimeter geometric accuracy is required (e.g. <1 mm within a 10 cm radius and <2 mm within 20 cm of isocenter) (Glide-Hurst *et al* 2021). To enable reliable use of DWI in functional IGRT, such as identifying tumor subregions for dose painting, it must therefore achieve geometric accuracy comparable to anatomic MRI sequences such as *T*_1_- or *T*_2_-weighted imaging.

##### Current and future challenges

*Challenge 1: Hardware of MR-LINAC system*

The integration of MRI and LINAC components into a single, non-separable configuration in current commercial MR-LINAC systems introduces unique engineering challenges. These challenges stem largely from the need to accommodate both high-precision imaging and therapeutic radiation delivery within a limited physical footprint, resulting in hardware trade-offs that impact imaging performance.

(a) *Gradient system performance.* To allow passage of the radiation beam, MR-LINAC systems utilize split gradient coil design (Raaymakers *et al* 2009, Mutic and Dempsey 2014), creating a physical gap around the MRI isocenter. This architectural compromise weakens the gradient performance. The 1.5 T Unity MR-LINAC system has a maximum gradient strength of 34 mT m^−1^ and a slew rate of 120 T m s^−1^, however, under clinical operation, the system typically uses a maximum gradient strength of 15 mT m^−1^ and a slew rate of 65 T m s^−1^ (Kooreman *et al* 2020, Powers *et al* 2022). The maximum dynamic gradient strength on the 0.35 T MRIdian MR-LINAC system is 18 mT m^−1^, with a maximum slew rate of 200 T m s^−1^ (Kluter 2019). The inferior gradient performance hinders the implementation of DWI and other advanced quantitative MRI techniques, which rely on rapid and strong gradient switching for sensitivity to tissue microstructure.
(b) *Main magnetic field (B_0_) stability.* The LINAC gantry where the radiation generation and delivery components are mounted, is surrounded by high-permeability mu-metal shielding to minimize magnetic interference (Whelan *et al* 2018), however, this introduces strong electromagnetic coupling with the *B*_0_ field. During gantry rotation, this coupling induces substantial eddy currents and dynamic center-frequency shifts (on the order of ±400 Hz), resulting in distortion and artifacts in MR imaging on the 0.35 T MRI-LINAC (Curcuru *et al* 2022).

Together, these limitations related to gradient performance and B_0_ field stability impose fundamental constraints on MR-LINAC image quality. These effects disrupt the spatial uniformity of the applied diffusion gradients, leading to spatially varying and poorly calibrated *b*-values. As a result, quantitative diffusion metrics such as the ADC may become biased or inaccurate, especially in regions farther from isocenter (McDonald *et al* 2023). The Elekta Unity consortium has recommended that accurate ADC values with less than 5% deviation from the ground truth can be achieved within a radius of 7 cm of the isocenter.

*Challenge 2: RF coil configurations*

To support accurate treatment planning, MR-simulator systems are equipped with RT-specific positioning devices, such as flat tabletops, indexed immobilization masks, and coil holders, to enable consistent and reproducible patient setup between simulation and treatment (Moore-Palhares *et al* 2023). However, the use of rigid immobilization devices, while critical for setup reproducibility, imposes significant limitations on coil placement and patient positioning flexibility (Rostami *et al* 2024). Unlike conventional MRI scanners, where flexible surface coils can be positioned close enough to the body to maximize SNR, RT setups often require a greater distance between the surface coils and the patient to avoid body surface deformation—an essential consideration for accurate RT planning and delivery. Additionally, support devices such as cradles and vacuum bags further increase the separation between the anatomy and receiver coils, resulting in further SNR degradation (Mandija *et al* 2019).

These challenges are even more pronounced in MR-LINAC systems. To avoid interfering with the treatment beam, RT coils used in MR-LINACs must be radiotranslucent, which limits coil design options (the materials and the number of coil channels). These coils typically employ low-conductivity materials and sparse geometries with open apertures to minimize beam attenuation. They are also often mounted on coil holders that increases the distance between the coil and the patient’s body. As a result, MR-LINAC systems generally exhibit lower SNR compared to both diagnostic MRI and MR-simulator systems, making it even more challenging to achieve the image quality required for advanced imaging applications such as DWI.

*Challenge 3: DWI pulse sequence options*

Despite single-shot EPI is robust to motion, it suffers from image distortion, chemical shift artifacts, and relatively low spatial resolution (typically around 2 × 2 × 5 mm^3^ on diagnostic MRI and 4 × 4 × 4 mm^3^ on MR-LINAC). To address these challenges, multi-shot segmented EPI techniques divide k-space acquisition into multiple shorter segments, reducing echo train length and thereby decreasing spatial distortion such as RESOLVE (Readout Segmentation Of Long Variable Echo trains) (Porter and Heidemann 2009). However, multi-shot acquisitions are more susceptible to inter-shot motion, which can introduce phase inconsistencies between segments, resulting in blurring or ghosting artifacts (Dai *et al* 2018, Moulin *et al* 2025). When RESOLVE is used with a reduced field-of-view (FOV) (e.g. Siemens ‘ZOOMit-RESOLVE’), a 2D spatially selective RF pulse that excites only a limited region in the phase-encoding direction, thereby minimizing distortion and blurring (Rieseberg *et al* 2002, Riffel *et al* 2014). However, this approach substantially reduces the overall SNR of the acquired images (Jeong *et al* 2005). To mitigate these limitations, acceleration techniques such as simultaneous multislice imaging (Zahneisen *et al* 2015) have been incorporated to shorten scan time. Additionally, advanced reconstruction method such as image reconstruction by iterative sampling (Jeong *et al* 2013), multiplexed sensitivity encoding (Chang *et al* 2015), and retrospective 3D rigid motion correction using shot-to-shot navigators (Riedel Ne Steinhoff *et al* 2021), have been developed to reduce noise, improve SNR, and correct for motion artifacts. Nevertheless, these advanced segmented DWI techniques are only available on certain diagnostic MRI and MR-simulator scanners and not yet available on any commercial MR-LINAC system, limiting their immediate clinical translation in MRIgRT.

Alternatively, DW-turbo spin echo (TSE) sequences uses Cartesian or overlapping radial k-space trajectories (e.g. PROPELLER—periodically rotated overlapping parallel lines with enhanced reconstruction) to address some of inherent limitations of conventional DW-EPI sequences (Deng *et al* 2006, 2008b, 2009). EPI readouts are *T*_2_*-weighted, making them more sensitive to field inhomogeneity and susceptibility-induced signal loss, whereas the 180° refocusing pulses and *T*_2_-weighted signal decay in TSE readouts leading to less distortion. These features make DW-TSE a promising alternative for RT applications, where geometric accuracy is critical. However, DW-TSE sequences exhibit lower SNR—primarily due to longer echo times (TE) and require longer scan times compared to single-shot EPI, making them more vulnerable to motion artifacts. While DW-PROPELLER inherently provides some motion correction through its rotating, overlapping radial blade acquisition, this k-space redundancy further extends scan duration, and any misregistration between blades can introduce image artifacts. An important technical limitation of DW-TSE is the violation of the Carr–Purcell–Meiboom–Gill (CPMG) condition when strong diffusion gradients are applied. This violation can cause phase dispersion due to inconsistent refocusing of primary and stimulated echoes, resulting in signal loss and bias in ADC estimations (Zhang *et al* 2020). To mitigate CPMG violations in DW-TSE, DW-SPLICE separates the acquisition of fast spin-echo signals (Deng *et al* 2008a); by combining the signals from these two echo pathways, non-CPMG artifacts are effectively cancelled and the SNRs of the DW images are improved. DW-SPLICE has been implemented on some diagnostic MRI and MR-simulator scanners, and more recently, on the 1.5 T Unity MR-LINACs DW-SPLICE on the MR-LINAC generally outperformed single-shot EPI and TSE in terms of repeatability and reproducibility for head and neck DWI prior to RT (McDonald *et al* 2023). Nonetheless, SNR enhancement, acquisition of more *b*-values, and tests on other body sites are warranted to enable clinical deployment of DW-SPLICE for MRIgRT.

*Challenge 4: DWI parameters on MR-LINAC*

Due to hardware constraints—particularly limited gradient amplitude and slew rate—achieving the same effective diffusion time (Δ_diff_) on MR-LINACs typically result in reduced *b*-values. In addition, to compensate for low SNR at higher *b*-values (e.g. 1000 s mm^−2^), multiple signal averages are acquired (up to 10), substantially increasing scan time. While averaging can reduce random noise, it does not correct for ADC measurement bias caused by Rician noise if performed in the magnitude domain (Pfaff *et al* 2024). Therefore, low SNR, especially at high *b*-values, remains a major limitation in DWI on MR-. It compromises the stability of derived diffusion metrics, propagates error through non-linear model fitting, and reduces reproducibility (Hutchinson *et al* 2017, Kooreman *et al* 2020). To balance image quality, quantification accuracy, and scan time, the Elekta Unity consortium recommends limiting the maximum *b*-value at 500 s mm^−2^ (Kooreman *et al* 2020). However, this limitation restricts the use of advanced diffusion biophysical models (introduced in section 3 and detailed in chapter II), which require a broader and higher range of *b*-values to probe tissue microstructures.

Although cross-platform calibrations using diffusion phantoms have been made across diagnostic MRI, MR simulators, and MR-LINACs (section 11), significant hardware-related discrepancies remain. Differences in gradient performance, coil configurations, and system-specific signal behavior introduce variability in DWI-derived metrics. These inconsistencies hinder harmonization of diffusion parameters and challenge the reliable detection of subtle changes critical for early response assessment for BIgART (Leibfarth *et al* 2018).

##### Advances in science and technology to meet challenges

*Advancements in DWI image quality enhancement*

Image quality and signal fidelity are the premises for accurate quantification of tumor tissue properties using DWI. A key goal for future improvement is to reduce distortion of EPI-based DWI so that tumor contours defined on high-resolution anatomic images can be accurately transferred to corresponding DWI images for reliable subregional functional assessment. Recent deep learning (DL)-based approaches have shown promise in distortion correction by synthesizing distortion-free DWI images from anatomical images, and applying deformable registration to correct distortion in acquired DWI (Schilling *et al* 2019, Hu *et al* 2020, Yu *et al* 2023). However, these supervised models require large, high-quality, distortion-free DWI datasets as ground truth for training, which are currently lacking on MR-LINACs. Moreover, supervised learning models are susceptible to domain shift and limited generalizability across different MRI platforms (Zhou *et al* 2023). To overcome these challenges, future efforts may focus on developing self-supervised and unsupervised learning techniques for distortion correction (Zimmer *et al* 2024, Liu *et al* 2025).

As an alternative to EPI-based sequences, distortion-less DW-SPLICE suffers from inherently low SNR, limiting the achievable *b*-values for advanced diffusion modeling. Traditional denoising methods, such as Marchenko–Pastur principal component analysis (PCA), require more than 30 diffusion directions per voxel, which is not feasible in time-limited clinical settings (Veraart *et al* 2016a). While simple spatial smoothing filters can improve SNR, they often blur anatomical details. DL-based denoising methods offer a potential alternative but depends on large, well-curated training datasets, and risk introducing hallucinated features or artifacts in the absence of strong priors (Chen *et al* 2023). Beyond improving image quality, several acquisition-specific factors must be addressed to optimize DWI protocols on MR-LINAC systems. First, spatially varying deviations in effective *b*-values caused by gradient nonlinearity and magnetic field imperfections must be corrected to ensure quantitative accuracy (Lee *et al* 2020). Second, the longer Δ_diff_ on MR-LINAC systems must be carefully considered, as it affects diffusion-time-dependent parameters and may influence the interpretation of tissue microstructure.

*Advancements in hardware systems*

(a) Next generation MR-LINAC systems. In addition to Unity (1.5 T) and MRIdian (0.35 T), two emerging MR-LINAC platforms are in various stages, each offering different magnet strength, image performance, and dosimetric behavior. One such system is Aurora-RT (MagnetTx Oncology Solutions, Canada) (Fallone *et al* 2009), a 0.5 T MR-LINAC currently gaining regulatory approvals and transitioning into clinical use. It integrates a 6 MV LINAC with a bi-planar superconducting 0.5 T MRI magnet in a parallel beam configuration, eliminating electron return and streaming effects common seen in perpendicular-field systems. Aurora-RT features the largest bore (110 × 60 cm) and ±25 cm lateral couch shifts, making it suitable for treating more anatomical sites. Another system is the Australian MR-LINAC prototype (Keall *et al* 2014), which combines a custom-built open-bore 1.0 T MRI where a 6 MV radiation beam orientation is fixed with respect to the magnet (Jelen *et al* 2020).
  These systems introduce novel magnet geometries, flexible beam paths, and improved integration. While they promise broader clinical applications and workflow improvements, their capability to support high-quality DWI remains to be validated. As these technologies mature, future development should focus on hardware and system-level optimizations such as maintaining B0 uniformity during gantry motion, enhancing gradient performance and linearity, and incorporating active shielding.
(b) RF coil development for MRIgRT systems. Radiation-compatible, lightweight, and high-channel-count coils improve SNR while minimizing interference with the treatment beam. Integrated transmit-receive birdcage coils provide radiation transparency and eliminate the need for bulky surface arrays, preserving dose delivery accuracy (Dietrich *et al* 2024). Flexible ‘blanket’ coils that conform closely to patient anatomy enhance SNR employ lightweight, thin conductors and remote-tuning electronics, allowing for dense coil packaging without rigid hardware components, making them well-suited for MR-simulation and RT setups (Mengling *et al* 2021, Darnell *et al* 2022). High-impedance coil (HIC) arrays, which avoid lumped components in radiation paths, have also been developed. A prototype 32-channel radiolucent HIC receive array demonstrated improved SNR, reduced geometry factors, and minimal beam attenuation, highlighting its potential for MRIgRT applications (Zijlema *et al* 2020)

##### Concluding remarks

DWI acquired during MRI simulation and MR-LINAC treatment have the potential to enhance tumor targeting precision, support biological adaption, and personalize radiotherapy. However, the integration of DWI into MRIgRT, particular on MR-LINAC platforms, remains technically challenging due to hardware constraints such as limited gradient performance and suboptimal RF coil options, low SNR, geometric distortion, and suboptimal diffusion parameter settings. Overcoming these limitations will require a concerted effort in both software and hardware innovations. The advancements in DWI pulse sequences, acquisition protocol optimization, and denoising and distortion correction methods are premise for reliable quantitative imaging. Equally important are hardware advancements, including high-performance gradient and radiation-compatible RF systems. Furthermore, the complex interplay between scan efficiency, motion management, and image quality must be carefully balanced to ensure clinical feasibility.

### 2. DWI reconstruction methods for MRIgRT

#### Xun Jia

Department of Radiation Oncology and Molecular Radiation Sciences, Johns Hopkins University, Baltimore, MD, United States of America

##### Status

DWI reconstruction has been extensively studied over the years, with significant efforts aimed at achieving high image quality to enable the extraction of clinically useful information from minimal measurement data, thereby reducing data acquisition time. Many of the reconstruction techniques currently used in MRIgRT have followed from those developed for diagnostic MRI and methods specifically tailored for RT remain in the early stages. In this section, we begin with a general review of common DWI reconstruction techniques to provide background and context. Subsequent sections will narrow the focus to reconstruction strategies within the MRIgRT context, where clinical constraints and priorities differ from those in diagnostic imaging. For more comprehensive coverage of general DWI reconstruction, readers are referred to Shafieizargar *et al* (2023).

As mentioned in section 1, DWI acquisition involves measuring data at multiple *b*-values. In the case of fully sampled data, an image at each *b*-value can be reconstructed by applying an inverse Fourier transform to the measured *k*-space data. In multi-channel parallel imaging, this step is followed by combining the images from individual coil channels using known coil sensitivity maps, or maps that are jointly estimated during the reconstruction process.

Undersampling—whether in *k*-space or data averaging—is commonly used to reduce scan time but results in artifacts such as aliasing and excessive noise, both of which can compromise data fitting accuracy. To address these issues, reconstruction is often formulated as an optimization problem, where a data fidelity term enforces consistency between reconstructed images and measured signals. Regularization is typically applied to mitigate the effects of undersampling by incorporating prior information, guiding the selection of a preferred solution among many possible candidate solutions for the measured data. A prominent method in this category is compressed sensing, which assumes the solution is sparse in a transformed domain, with gradient-like operators such as Total Variation and wavelets used to emphasize image edges (Lustig *et al* 2007).

In DWI, the images at different *b*-values share common information, which can be exploited to enhance image reconstruction. For example, the similarity between images allows for low-rank regularization on the matrix formed with its column vectors corresponding to these images. The low-rank constraint is often expressed as minimizing the matrix nuclear norms. This approach has evolved into low-rank plus sparsity models to account for deviations from the low-rank assumption. While it is the subsequent step after image reconstruction to estimate DWI parameters from the reconstructed images for clinically relevant information, advanced reconstruction approaches combine image reconstruction with parameter estimation, allowing signal models to act as implicit regularizers.

A more effective way of employing prior information is to learn the image prior directly from image data used in routine clinical practice. Such prior knowledge may include image intensity distributions, tissue properties, anatomical structures, and other contextual features. Dictionary type regularization is a popular category, whereby the solution is expressed as a sparse representation of a dictionary learnt from image data. This approach effectively constraints the solution to a low-dimensional linear sub-space of the solution space formed by pixel values as the coordinate. In recent years, DL has demonstrated its power in medical image processing (Shen *et al* 2020), including for DWI reconstruction (Knoll *et al* 2020). It outperforms traditional techniques by learning the complex subspace structure of solutions, generally non-linear, and applying it to regularize solutions.

The efficiency of solving the reconstruction problem is crucial for practicality considerations in a clinical setting. Novel methods like the alternating direction method of multipliers (ADMMs) have been developed to accelerate convergence while handling those complex optimization models, such as non-differentiability. Recently, DL-based numerical algorithms have shown promise. These algorithms are often motivated by the classical numerical algorithm, such as ADMM, but trained to embed image regularization effects in the iterations, e.g. ADMM-NET (Yang *et al* 2020). Additionally, end-to-end DL models can directly map k-space data to reconstructed images without iterative processes (Zhu *et al* 2018).

##### Current and future challenges

Enhancing quantitative accuracy, encompassing both intensity fidelity and geometric precision, is a central goal of DWI reconstruction. In MRIgRT, the challenge to achieve this goal is compounded by several factors, many of which stem from hardware and protocol limitations as presented in section 1. Advanced reconstruction methods are needed to overcome the challenge associated with its relatively low SNR. In MR-LINAC systems, additional constraints further complicate the reconstruction process. This is further exacerbated by the difficulty to reduce acquisition time due to the long diffusion times needed under the low gradient strength. The long scan time also increases the likelihood of patient motion, which compromises validity of DWI models and hence challenges data analysis. Time constraints, especially during pre-treatment online imaging, also limit both the SNR and completeness of the acquired data used for reconstruction.

Longitudinal imaging applications, from treatment simulation through repeated imaging during the course of RT on the MR-LINAC systems, present unique demands. Ensuring consistency in DWI reconstructions across different protocols and different scanners, i.e. MR simulator and MR-LINAC, is critical for reliable clinical decision-making.

Another practical concern is the speed of solving the reconstruction problem. Iterative reconstruction methods often require careful hyperparameter tuning, a time-consuming process. While DL-based end-to-end models offer faster reconstructions, they come with their own set of challenges, including the need for large, high-quality training datasets, robustness to domain shifts (Antun *et al* 2020), and clinical explainability.

##### Advances in science and technology to meet challenges

To overcome the hardware and protocol-related limitations in RT-specific DWI reconstructions, continued research into advanced reconstruction algorithms is essential. Generally, to compensate missing information or correct error in the measured data, additional information is necessary. This supplemental information may come from accurate physics-based modeling of the DWI acquisition or by leveraging prior knowledge extracted from existing clinical images acquired in routine practice. Model-based reconstructions can be enhanced by incorporating more precisely the physics of the data acquisition process. Data-driven DL-type reconstruction approaches offer the ability to extract useful prior information from existing imaging data to guide and improve reconstruction. However, further development is needed to address key challenges associated with DL-based techniques, including robustness, generalizability, and interpretability. A hybrid approach that combines data-driven DL methods with model-driven techniques may leverage the strengths of both.

Prior information can exist at various levels, each with differing degrees of specificity and validity. The effectiveness of such information in reconstruction tasks depends on how well it aligns with the specific clinical context. Patient-generic priors, such as population-averaged anatomical templates or statistical models, can provide useful guidance but often lack the specificity needed for accurate reconstruction in individual patients. In contrast, patient-specific priors offer detailed anatomical information tailored to the individual, making them particularly effective for personalized reconstruction (Grandinetti *et al* 2022). However, such priors are inherently limited to the individual patient and may misguide reconstructions when applied to others. In the context of MRIgRT, one benefit is the routine availability of patient-specific prior imaging information. As treatment on MR-LINAC progresses, additional patient-specific data become available, enabling adaptive strategies such as dynamically adjusting imaging protocols to reduce acquisition time or dose while preserving accuracy (Yan *et al* 2013). While patient-specific priors are often more effective than generic ones, their validity must be carefully assessed to ensure appropriate use and to avoid introducing bias into the reconstruction process.

From the MRIgRT clinical application perspective, the speed of reconstruction is a priority. This is not only to shorten the imaging time (see section 1) for online adaptive treatment procedure, but also to streamline the process, to reduce the chance of patient motion that may impede or invalidate imaging information for image guidance, and to allow advanced data analysis to decipher clinically valuable information. Improvements in numerical algorithms and DL-based methods will help accelerate both reconstruction and data analysis, allowing the extraction of clinically relevant information rapidly. To optimize hyperparameter selection for reconstruction, building the relationship between optimal parameters with patient/scan scenarios, or novel methods such as reinforcement learning could be employed (Shen *et al* 2018).

For DWI applications in MRIgRT, studies are needed to endow the DWI results with clinically relevant information, for instance, tumor cell death, microvasculature destruction, and immune response, etc. Stability and robustness of the DWI-derived metrics are essential for clinical applications, particularly in longitudinal studies across different scanners. Developing reconstruction methods should bear these desires in mind for informed clinical decision making. Finally, as DWI becomes more integrated into the RT workflows, objective benchmarks are essential to evaluate algorithm performance. Initiatives like reconstruction competitions organized by the scientific community can provide a common ground for comparing reconstruction methods.

##### Concluding Remarks

In conclusion, this chapter provides a roadmap for DWI reconstruction in MRIgRT, a field in its early stage but with great potential. While DWI techniques have been extensively studied in diagnostic imaging, their application in RT requires targeted solutions to address unique issues in this context, such as low SNR, longitudinal data analysis, and integration with RT workflows.

### Acknowledgements

We would like to acknowledge the support from the National Institutes of Health (Grants # R37CA214639, R01EB032716, R01CA227289).

### 3. DWI model fitting for MRIgRT

#### Jiaren Zou

Department of Radiation Oncology, University of Michigan, Ann Arbor, MI, United States of America

##### Status

The number of DWI data acquisitions is limited by the complexity of diffusion-sensitizing schemes, extended scan time, and low SNR—challenges that are particularly pronounced on MR-LINAC systems. These constrains impact the accuracy and robustness of biophysical model-based parameter estimation. Therefore, the choice of signal representation or diffusion model and its associated fitting method is critical to ensure reliable interpretation and effective clinical applications.

One approach to interpret DWI data is through signal representation, which makes minimal assumptions about tissue properties (Novikov *et al* 2018). For instance, cumulant expansion represents the logarithm of the diffusion signal as a Taylor series in terms of the *b*-value. The commonly used ADC is calculated by fitting diffusion signal to the cumulant expansion up to the first order of *b*-values. To obtain more specific characterization of tissue anisotropy and heterogeneity, advanced techniques have been developed. To better capture the non-Gaussian diffusion components present in disordered tissue microstructure, DKI represents the signal to the second order of *b*-values. Open-source tools are available for model fitting of DKI with various fitting algorithms (Henriques *et al* 2021). In tumor tissue, water diffusion is generally isotropic, diffusion signals are often modeled with assumptions about the underlying microstructure (see chapter II for details on these models). However, robust model fitting of these advanced techniques is challenging due to the increased degrees of freedom in parameters fitting (Kiselev 2017).

##### Current and future challenges

Model degeneracy is a fundamental challenge due to the mismatch between insufficient data acquisition and high complexity of the underlying signal models or representations. Degeneracy refers to the existence of multiple parameter sets that can fit the acquired signal equally well, leading to ambiguity in model fitting. This degeneracy can be explained and visualized through multiple local minima or the flatness of model fitting landscape (Jelescu *et al* 2020).

The challenge is particularly acute on MR-LINAC systems, where low SNR and limited *b*-value sampling further exacerbate degeneracy, resulting in numerous parameter combinations that may appear equally plausible. As a result, the derived biological metrics lack robustness and accuracy, making it difficult to distinguish real physiological variations from estimation uncertainty (Reynaud 2017). For instance, model fitting fails to determine five model parameters of a simple two-compartment model of neuronal tissue from over 60 measurement point under practical noise levels (Jelescu *et al* 2020). In tumor microstructural parameter mapping, detecting variations of intracellular volume fraction <3% and cell radius <0.5 $\mu {\mathrm{m}}$ were found to be unreasonable through simulations under typical *in vivo* SNR (Reynaud 2017). Resolving model degeneracy is, therefore, a central objective in the development of advanced DWI model fitting methods.

One common approach to mitigate degeneracy involves incorporating prior knowledge to constrain the parameter space. Several studies have introduced biophysical constraints or prior distributions to reduce the degrees of freedom in model fitting and improve fitting stability. However, when these priors deviate from actual tissue properties, they can introduce systematic bias to the estimates (Jelescu *et al* 2020). For example, using different sets of constraints in model fitting resulted in non-negligible differences in neurite signal fractions in the human brain (Jelescu *et al* 2020). In addition, prior knowledge specific to particular disease states or tissue types may not generalize across broader applications. Each diffusion signal model often imposes unique parameter constraints, making direct comparison across models difficult.

Another challenge is the computational burden associated with fitting advanced, nonlinear diffusion models. Nonlinear least squares fitting is commonly used (Jelescu *et al* 2020), which typically minimize a residual loss function to estimate model parameters. However, these loss functions are often insensitive to variations in model parameters (Transtrum *et al* 2011, Lavielle and Aarons 2016). It is also computationally intensive and highly susceptible to bias and uncertainty in the presence of noise. Strong collinearity among parameters further complicates the estimation process (Box 1960, Machta *et al* 2013). This becomes a critical limitation in MRIgRT, where timely treatment plan adaptation is essential. Therefore, the development of fast, robust model-fitting algorithms is highly desirable to ensure that quantitative imaging can be seamlessly integrated into the clinical workflow of MR-LINAC treatment.

##### Advances in science and technology to meet challenges

Dictionary-based model fitting methods, such as dictionary learning or matching, can accelerate and improve the accuracy of diffusion model fitting. These approaches exploit the inherent sparsity of DWI signals to better identify the underlying signal and model parameters. A widely used example is accelerated microstructure imaging via convex optimization (Daducci *et al* 2015), which transforms complex non-linear fitting problems into linear formulations. Numerical simulations are employed to generate a dictionary of synthetic diffusion signals, representing a range of possible tissue microstructural properties. The observed DWI signals are then matched to this dictionary to estimate the corresponding model parameters. This approach offers several advantages: it enables rapid parameter estimation, making it well-suited for processing large datasets and real-time applications of MRIgRT. It also allows incorporation of spatial regularization in parameter estimation, which enhances precision and visual quality of the parametric maps. However, these methods can also introduce bias into the parameter estimates, particularly if the dictionary does not accurately capture the signal characteristics of tissue properties. Moreover, the generalizability of dictionary-based methods is limited, as a new dictionary, or different spatial constraints, need to be devised for a new acquisition protocol, tissue type, diffusion model, or signal representation.

DL-based methods have shown great promise in improving the accuracy, precision, and speed of model fitting compared to conventional methods. Instead of using manually defined prior distributions for model parameters, deep networks can learn more realistic priors directly from large datasets, capturing complex relationships between DWI signals and model parameters. Additionally, DL networks can learn sparse, nonlinear representations of DWI signals, which enables accelerated model fitting. A commonly used framework is supervised DL, in which neural networks are trained to map input DWI signals to model parameters using a large dataset of simulated signal-parameter pairs (Barbieri *et al* 2020). The weights of the neural networks are optimized to minimize the estimation error on the training dataset in the training phase. The weights are then fixed, and the network is applied to unseen *in vivo* DWI signals for rapid parameter estimation. In simulations, these methods substantially reduced the root mean squared error of IVIM model parameters compared to conventional least squares fitting (Barbieri *et al* 2020). Supervised DL further provided a modest improvement in root mean squared error compared to Bayesian methods, while also achieving a fitting speed several orders of magnitude faster than both least squares and Bayesian approaches. However, since high quality *in vivo* ground truth parameter maps are rarely available, especially for complex or nonlinear models, supervised learning often relied on training data generated from manually designed parameter distributions. This reliance in turn can introduce bias and limit the generalizability of supervised learning-trained models (Gyori *et al* 2022).

To address these limitations, self-supervised learning framework has been developed. Such methods (Stehling *et al* 1991) use the acquired DWI data from a patient cohort to train the network specific to that population. Instead of minimizing error between predicted and ground truth parameters, self-supervised models minimize the difference between the acquired DWI signal and the predicted signal reconstructed from estimated parameters (Sen *et al* 2024). This allows the network to learn implicit population-level priors directly from the data. However, the reliability of these methods depends on whether the available training data adequately represent the true population distribution. Finding optimal strategies to learn population-level prior knowledge and incorporate it into model fitting remains an open and promising area for future research.

Other developments include approaches that estimate model parameters directly from signal moments or cumulants (Golkov *et al* 2016). These methods enable significant linearization of the nonlinear problems, substantially simplifying parameter estimation and reducing computational complexity. Practically, it allows accurate parameter estimation at lower *b*-values and shorter acquisition times, which are more easily achievable with clinical MR scanners. However, the difficulty in accurately and precisely estimating the cumulants presents a limitation of this method. In parallel, advanced optimization algorithms have been investigated. Recent studies have shown that gradient-free optimization algorithms can outperform traditional gradient-based algorithms in terms of runtime, fitting accuracy, and precision for commonly used biophysical diffusion models in the brain (Jelescu *et al* 2020).

##### Concluding remarks

Model fitting in DWI is challenging due to sparse data acquisition, low SNR, and complexity of biophysical signal models or representations. A variety of strategies have been proposed to address this challenge, leveraging different forms of prior knowledge and fitting algorithms. While incorporating prior knowledge can help stabilize fitting and reduce model degeneracy, it is important to evaluate the validity of prior knowledge and potential bias in specific applications. An important open question is how to effectively acquire or learn reliable prior knowledge and incorporate it into model fitting and to validate the accuracy of the estimated model parameters. Although priors can alleviate model degeneracy and improve estimation robustness, further efforts are needed to improve DWI scan efficiency, especially on platforms like MR-LINACs where acquisition constraints are more pronounced. Finally, transparent code sharing and open-source development of model fitting pipelines are essential for facilitating reproducibility, comparative benchmarking, and continued innovation in this field.

#### Concluding statement for chapter I

*Jie Deng*

Department of Radiation Oncology, University of Texas Southwestern Medical Center, Dallas, TX, United States of America

This chapter provides an overview of the three general components of DWI in the context of radiotherapy: acquisition protocols, image reconstruction, and signal model fitting. Compared with conventional MRI systems (1.5 T and 3.0 T), DWI on MRI simulators, despite using similar field strengths, faces notable challenges due to specialized RT configurations. These challenges are even more pronounced on MR-LINAC systems (0.35 T and 1.5 T), where hardware constraints such as *B*_0_ inhomogeneity, reduced gradient strength, low SNR, increased artifact levels, and limited coil configurations further degrade image quality. In addition to hardware-related limitations, challenges in DWI signal model fitting, such as model degeneracy and fitting instability, exist across all MRI platforms but are especially problematic on MR-LINAC systems due to inferior image quality. Standardization and harmonization of DWI acquisition and processing between MRI simulators and MR-LINAC platforms is essential to enable confident and consistent longitudinal tracking of treatment-induced changes. Improving the speed of DWI acquisition, enhancing reconstruction quality, and increasing the robustness and accuracy of model fitting are critical steps toward successful integration of DWI into both MRI simulation workflows and MR-LINAC ART. Note that harmonization should proceed only after confirming the biophysical validity, robustness, and transferability of the methods across platforms, field strengths, and clinical contexts. Alternatively, rather than relying on biomarkers from direct model fitting, which often exhibits large fitting uncertainties under clinically achievable SNRs, it maybe be more effective to generate robust, coarse-grained metrics. Although these metrics may lack direct biological interpretability, they can reliably capture relative changes or characterize distinct trends in tumor response throughout the course of radiotherapy. Establishing their biological relevance will require further studies correlating these surrogate measures with physiologically meaningful endpoints.

## Chapter II. Exploring tumor microenvironment with DWI from MRI scientist’s perspective

### 4. DWI for tissue microstructure

#### Christopher C Conlin^1^, Anna M Dornisch^2^ and Tyler M Seibert^1,2,3,4^

^1^ Department of Radiology, University of California San Diego Health, La Jolla, CA, United States of America

^2^ Department of Radiation Medicine and Applied Sciences, University of California San Diego Health, La Jolla, CA, United States of America

^3^ Department of Urology, University of California San Diego Health, La Jolla, CA, United States of America

^4^ Department of Bioengineering, University of California San Diego Jacobs School of Engineering, La Jolla, CA, United States of America

##### Status

While ADC derived from DWI is typically a gross oversimplification of underlying tissue microstructure, it remains the principal quantitative measure of diffusion for evaluating tumors in the body, including the prostate, breast, endometrium, and cervix (Mann *et al* 2019, Turkbey *et al* 2019, Maheshwari *et al* 2022). Diffusion tensor imaging (DTI) is the extension of the ADC concept to three dimensions. By applying diffusion encoding gradients in at least 6 non-colinear directions, it is possible to estimate a tensor of ADC values which characterize a 3D ellipsoid. The orientation and magnitude of the axes of this ellipsoid define the principal directions of diffusion and the degree of diffusional anisotropy within the underlying tissue (Basser *et al* 1994, Hiltunen *et al* 2005). A key parameter derived from the ADC tensor is fractional anisotropy, which quantifies the average anisotropy of the tissue, and ranges from 0 (completely isotropic diffusion) to 1 (completely directional diffusion) (Jeon *et al* 2018). The directional information from the ADC tensor is the foundation of tractography, which can be used to generate 3D maps of structured tissue like neural white matter tracts or renal pyramids (Cage *et al* 2015). Important to radiation oncologists, DTI assessments of neuronal microstructure may provide important prognostic indicators of cognitive decline (Tringale *et al* 2019). Tractography-informed RT is currently being evaluated prospectively (ClinicalTrials.gov ID NCT04343157) as a means to mitigate neurologic morbidity due to radiation toxicity.

##### Current and future challenges

*Limitations of the ADC and DTI models*

The main limitation of the ADC and DTI models is its strict assumption of a single compartment of Gaussian diffusion. This leads to an inability to resolve multiple diffusion microenvironments (modes of diffusion) within a single voxel, as the signals from the different microenvironments are merged into a single ensemble average. For example, ADC values of highly cellular tumors in the brain (glioblastoma and primary central neural system lymphoma) rarely fall below that of normal appearing white matter (Maier *et al* 2010). Higher than expected ADC values for these tumors can be attributed to the presence of edema and necrosis, which result in more free water diffusion and yield an increase in ADC that offsets the ADC reduction imposed by hyperplasia (Chenevert *et al* 2006). Similarly, the ability to resolve individual neural fibers in DTI is commonly lost due to edema or the presence of multiple fiber orientations that cross within a single voxel (McDonald *et al* 2013). Furthermore, these models are fundamentally unable to characterize non-Gaussian diffusion, like the restricted diffusion observed within intracellular compartments (Hope *et al* 2016). Characterization of restricted intracellular diffusion allows for reliable estimation of tumor cellularity, an important prognostic indicator (Yamin *et al* 2016).

##### Advances in science and technology to meet challenges

*Advances in ADC and DTI models* a more extensive image acquisition approach, that varies the magnitude and orientation of diffusion weighting, enables the separation of signal from different microstructural compartments within a single voxel (Basser 2002, Assaf and Basser 2005, Wedeen *et al* 2005, Alexander *et al* 2010, White *et al* 2013). Incorporation of strong diffusion weighting (*b*-values >1500 s mm^−2^), in particular, has demonstrated improved sensitivity to the restricted diffusion signals from highly cellular structures like tumors. While all advanced DWI techniques vary the magnitude and orientation of diffusion weighting, they differ in implementation details and how the diffusion signal is modeled. Diffusion spectrum imaging, for example, is a model-free approach that relies on a dense sampling of diffusion magnitudes and orientations to reconstruct an arbitrarily complex diffusion microenvironment (Van *et al* 2010). The long scan time required for such dense sampling is impractical for clinical application, however, so techniques like q-ball imaging are generally employed which trade a reduction in scan time for a reduction in the ability to resolve different aspects of the diffusion microenvironment (Tuch 2004).

**Figure 2.**
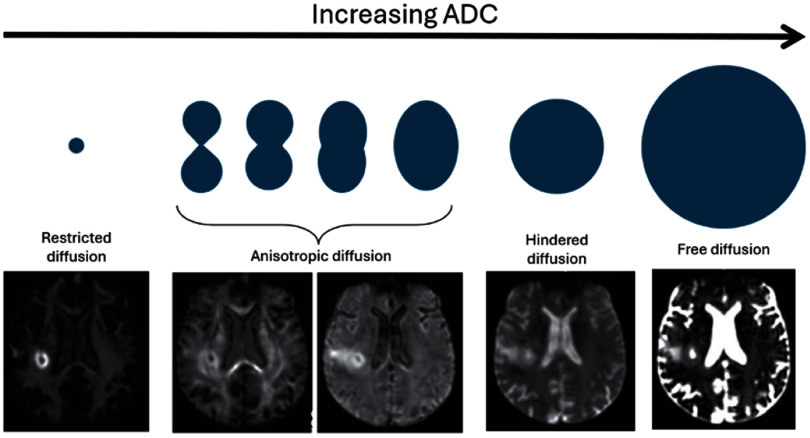
RSI for brain imaging. The different RSI compartments use spherical harmonic basis functions (blue) account for both the scale and orientation of diffusion. Compartments are presented in order of increasing ADC. Images showing the relative proportion of signal from different compartments are presented for a patient with a brain tumor (glioblastoma, bright ring on the restricted signal image).

RSI is a promising technique that imposes minimal assumptions about the underlying tissue microstructure while also affording a computationally efficient implementation suitable for routine clinical application (White *et al* 2013). Acquisition times are clinically feasible on standard scanners and have already been incorporated into clinical workflows for several applications, including brain, prostate, and breast. In brief, RSI models the diffusion signal as being derived from a linear mixture of a finite number of compartments, each is characterized by fixed diffusion parameters. The total signal is the sum of the signal contributions from each compartment, which are weighted by the volume fraction occupied by each. By fixing the diffusional parameters of each compartment, solving for the signal contribution (weighting factor) from each compartment becomes a linear estimation problem that can be computed in seconds and is considerably more robust to image noise than nonlinear fitting strategies (White and Dale 2009). RSI for brain imaging describes each compartment using spherical harmonic basis functions to account for both the scale and orientation of diffusion, with the scale ranging from restricted to free diffusion (figure 2):

\begin{equation*}S = \left[ {R\left( {D_{\mathrm{T}}^{\left( 1 \right)}} \right){Y_{{\mathrm{L1}}}} \ldots { }R\left( {D_{\mathrm{T}}^{\left( J \right)}} \right){Y_{{\mathrm{L}}J}}{ }{{\mathrm{e}}^{ - b{D_{\mathrm{L}}}}}{ }{{\mathrm{e}}^{ - b{D_{\mathrm{F}}}}}} \right]{\left[ {{\beta ^{\left( 1 \right)}} \ldots { }{\beta ^{\left( J \right)}}{ }{\beta ^{\mathrm{L}}}{ }{\beta ^{\mathrm{F}}}} \right]^{\mathrm{T}}},\end{equation*}

where $S$ is the matrix of signal intensity values at each *b*-value and diffusion gradient direction. $R$ is called the ‘fiber response function’, which models the predicted signal attenuation due to a single neuronal fiber. $D_{\mathrm{T}}^{\left( 1 \right)} &lt; D_{\mathrm{T}}^{\left( 2 \right)} &lt; \ldots &lt; D_{\mathrm{T}}^{\left( J \right)}$ are the transverse diffusivities of $J$ axonal compartments, with orientation described by spherical harmonic basis functions $Y$. Two isotropic diffusion compartments are included, with diffusivities that describe the motion of water longitudinally through axons (${D_{\mathrm{L}}}$) and freely in cerebrospinal fluid, for example (${D_{\mathrm{F}}}$). The contribution of each compartment to the overall signal is indicated by the weighting factors ${\beta ^{\left( i \right)}}$.

For tissues where diffusion is largely isotropic (e.g. breast or prostate), the RSI model can be reduced to a superposition of Gaussian compartments with different ADC values. An isotropic three-compartment model has been proposed for breast DWI, and a four-compartment model has proven valuable for diagnosing and characterizing prostate cancer (see chapter III section 10) (Conlin *et al* 2021, Feng *et al* 2021, Rodríguez-Soto *et al* 2022). The model is described as:

\begin{equation*}S\left( b \right) = {C_1}{{\mathrm{e}}^{ - b{D_1}}} + {C_2}{{\mathrm{e}}^{ - b{D_2}}} + {C_3}{{\mathrm{e}}^{ - b{D_3}}} + {C_4}{{\mathrm{e}}^{ - b{D_4}}}\end{equation*}

where *S*(*b*) denotes the DWI signal at a particular *b*-value, ${C_i}$ is the unit-less weighting factor that describes the contribution of a particular compartment to the overall signal, and ${D_i}$ is the ADC of a particular compartment. The compartmental ADC values for this model are fixed at predetermined values that were optimized for prostate DWI (Conlin *et al* 2021): ${D_1}$ = 1.0 × 10^−4^ mm^2^ s^−1^, ${D_2}$= 1.8 × 10^−4^ mm^2^ s^−1^, ${D_3}$ = 3.6 × 10^−4^ mm^2^ s^−1^, and ${D_4}$= 0.1 mm^2^ s^−1^; corresponding roughly to restricted, hindered, and free diffusion, with an additional consideration (${D_4}$) for rapid pseudo-diffusion (vascular flow).

##### Concluding remarks

With the development of RSI and the continued advancement of other imaging techniques, imaging-based *in vivo* characterization of tissue microstructures is now reality, allowing for clinical application in the setting of cancer staging and image-guided treatment. Most robustly studied in the central nervous system and prostate, future efforts should focus not only on ongoing multi-disciplinary validation of existing measures but also on expansion of RSI models in other organ systems.

### 5. DWI for microcirculation

#### Sirisha Tadimalla

Institute of Medical Physics, The University of Sydney, Sydney, Australia

##### Status

Microcirculation refers to the circulation of blood within the smallest vascular networks in tissues, operating on a scale of a few hundred microns. On the size and time scale of clinical DWI, blood flow in this complex, pseudo-random vascular network (i.e. perfusion) mimics a diffusion process and contributes to the attenuation of the DWI signal. The IVIM model describes the contributions of the perfusion and diffusion processes to the DWI signal via a bi-exponential decay as (Le Bihan *et al* 1988):

\begin{equation*}{\raise0.7ex\hbox{$S$} \!\mathord{\left/ {\vphantom {S {{S_0}}}}\right.} \!\lower0.7ex\hbox{${{S_0}}$}} = f{{\mathrm{e}}^{ - b{D^*}}} + \left( {1 - f} \right){{\mathrm{e}}^{ - b{\mathrm{ADC}}}}\end{equation*}

where *f* is the fractional blood volume, D* is the pseudo-diffusion coefficient, attributed predominantly to blood flow with a small contribution from water diffusion within the microvasculature and ADC is the apparent diffusion coefficient in the extravascular space.

In RT, IVIM model parameters have been investigated as prognostic or predictive biomarkers of treatment response in several cancers (Mesny *et al* 2024). Studies have also demonstrated the use of IVIM parameters to differentiate tumor recurrence from pseudo-progression and radiation necrosis (Detsky *et al* 2017, Li *et al* 2021). Additionally, there is growing interest in the assessment of early IVIM parameter changes in tumors during RT. This research is enhancing our understanding of the effects of various dose fractionation schemes on tumor response (Mahmood *et al* 2017) and leading development of IVIM DWI applications for biologically guided adaptive RT (Kooreman *et al* 2021). Radiomics analysis of IVIM parameter maps is increasingly being used to obtain information on intra-tumoral heterogeneity, which could improve prediction models of treatment response (Wang *et al* 2021c).

The IVIM concept has led to the creation of a consumption and supply-based hypoxia (CSH) model. This model generates a hypoxia score based on the IVIM parameters, ADC and *f*, where a decreased ADC reflects the higher cell density within the tumor and increased oxygen consumption and an increased *f* reflects increased blood vessel density in the tumor indicating an increased supply of oxygen to the tumor. Based on the CSH model, decreased ADC and decreased *f* are associated with tumor hypoxia (Hompland *et al* 2018). As tumor hypoxia is a key driver of radio-resistance, the CSH model has shown potential for predictive modeling of radiation treatment response and development of adaptive RT strategies (Skipar *et al* 2024). Moderate correlations between ADC and cell density and between *f* and blood vessel density, respectively, form the basis for the CSH model.

##### Current and future challenges

In principle, IVIM perfusion parameters are related to classical perfusion parameters obtained from dynamic contrast enhanced (DCE)-MRI or PET imaging. Specifically, *f* corresponds to blood volume, D* is inversely related to the mean transit time of an injected tracer, and the product fD* reflects blood flow (Le Bihan 2019). Several other studies have attempted to correlate IVIM parameters with histopathological characteristics and DCE-MRI parameters, with mixed results (Bisdas *et al* 2014, Kooreman *et al* 2022). It is important to remember that *f* and D* will be influenced by any source of incoherent motion in the DWI voxel, which can vary depending on body site as well as voxel size and partial volume effects. Non-capillary arterial and venous blood flow, pulsatile CSF flow, and renal tubular flow have all been shown to affect IVIM parameters (Bane *et al* 2016, Gambarota *et al* 2017, Wong *et al* 2018). Additionally, the IVIM model describes an overly simplified tissue microstructure, with a single extravascular compartment encompassing multiple environments for water diffusion. The model also ignores inter-compartmental exchange, restricted diffusion, and anisotropy within the vascular compartment. While corrections such as taking the hematocrit into account can be applied, physiological interpretation of IVIM parameters is challenging, and over-interpretation of changes in IVIM parameters during and after RT should be avoided.

Additionally, although perfusion and diffusion are intrinsic properties of tissues, measurements of IVIM parameters depend heavily on the DWI acquisition settings. For example, variations in IVIM parameter measurements can arise from differences in effective diffusion times (Δ_diff_) in DWI studies. The diffusion time dependence of ADC is well known; longer diffusion times allow water molecules to encounter more barriers to diffusion, leading to a lower measured ADC (Bourne *et al* 2017). In the case of the vascular compartment, blood can traverse and change several capillary segments during long diffusion times, thus increasing the pseudo-diffusion contribution (Wu and Zhang 2019). On clinical systems, the value for Δ_diff_ is automatically set by the scanner depending on the chosen maximum *b*-value based on the achievable amplitude and slew rate of the gradient coils. Consequently, the minimum TE achievable varies according to scanner hardware. The IVIM model neglects contribution of *T*_2_ relaxation in the vascular and extravascular compartments to the DWI signal attenuation. This means that *f* in the IVIM model as in equation (6) only corresponds to the vascular plasma fraction at very small TE, i.e. when *T*_2_ relaxation can be assumed negligible. On clinical MRI scanners, the minimum achievable TE ranges from 50 ms to 100 ms; on some MR-LINACs, this TE range can be achieved by limiting the maximum *b*-value to <600 s mm^−2^. For these TEs, differences in *T*_2_ relaxation between the compartments can become significant. Ignoring these differences can lead to overestimation of the fractional blood volume *f* (Lemke *et al* 2010). The TE dependence can be corrected by modifying the IVIM equation to include a separate *T*_2_ relaxation term for each compartment, for example, the extended *T*_2_-IVIM model (Jerome *et al* 2016). Corrected values for *f* can then be obtained using DWI images acquired at multiple TEs as well as multiple *b*-values, using optimized protocols to mitigate the expected increase in scan time.

Another key challenge for reliably estimating IVIM parameters is choosing appropriate *b*-values and fitting methods. Many clinical MRI scanners cannot acquire DWI with very low *b*-values (<50 s mm^−2^), leading to inaccurate effective *b*-values. If not corrected for, this complicates the estimation of IVIM perfusion parameters, particularly D*, and most applications of IVIM in RT estimate only *D* and *f* reliably. Precision of all IVIM parameters is further impacted by signal noise and the choice of the fitting method (Gurney-Champion *et al* 2018).

Additionally, standard EPI-based DWI is prone to geometric distortions and artifacts from breathing and cardiac motion further degrade image quality, making the associated IVIM parameter maps unsuitable for RT planning. For longitudinal assessment of IVIM parameters during and after RT, it is crucial to apply robust image co-registration during model-fitting (Li *et al* 2024), as well as account for repeatability and reproducibility errors in estimated IVIM parameters to ensure accurate evaluation of treatment response. To ensure comparable IVIM measurements, multi-site studies should harmonize DWI acquisition and processing methods across scanners (detailed in chapter IV, sections 11–13). For example, ensuring harmonization of diffusion times on clinical systems, rather than only TE, may allow for better comparisons of IVIM parameters across studies.

##### Advances in science and technology to meet challenges

Advancements in image acceleration, motion management, flow compensation and non-EPI-based readouts for DWI are expected to reduce noise and distortion artefacts, improving the reliability of IVIM parameter estimates (Yang *et al* 2023). DL-based image reconstruction techniques are emerging as solutions to quickly produce high quality DWI images and can potentially improve the accuracy of IVIM modelling (Hanamatsu *et al* 2023). Additionally, the use of physics-informed neural networks for efficient image denoising and model fitting is gaining traction (Kaandorp *et al* 2021). Furthermore, the IVIM model itself can be adapted to describe complex tissue microcirculation, for example, by merging the DTI and IVIM formalisms to account for directionality in the vascular compartment (Abdullah *et al* 2016). However, for these advancements to be clinically applicable, standardized protocols for both DWI acquisition and IVIM processing are crucial. The Open Science Initiative for Perfusion Imaging (OSIPI) is leading efforts to standardize post-processing for IVIM modeling (https://github.com/OSIPI/TF2.4_IVIM-MRI_CodeCollection). Participation of the RT research community in global consensus initiatives is essential for the successful integration of IVIM DWI into routine RT clinical practice.

##### Concluding remarks

The IVIM model is a potentially valuable tool for quantifying microcirculation in tumors and surrounding tissues, aiding in the prediction treatment response during and after RT. However, to fully realize the benefits of this method in RT, it is essential to address challenges relating to imaging acquisition and processing through global consensus and standardization.

### Acknowledgements

This work was supported in part by the Cancer Institute NSW Early Career Fellowship Grant CINSW/2022/ECF1462. The contributions and insightful discussions from Dr Yu-feng (Erin) Wang are sincerely acknowledged.

### 6. DWI for tissue heterogeneity

#### Muge Karaman^1,2^, Xiaohong Joe Zhou^1,2,3,4^

^1^ Center for Magnetic Resonance Research, University of Illinois at Chicago, Chicago, IL, United States of America

^2^ Department of Biomedical Engineering, University of Illinois at Chicago, Chicago, IL, United States of America

^3^ Department of Radiology, University of Illinois at Chicago, Chicago, IL, United States of America

^4^ Department of Neurosurgery, University of Illinois at Chicago, Chicago, IL, United States of America

##### Status

Tissue heterogeneity is key to cancer diagnosis and treatment assessment (Meacham and Morrison 2013), stemming from various factors such as genetics, epigenetics, physiology, and pathology, leading to structural intravoxel tissue heterogeneity. While histopathology remains the gold standard for assessing tissue composition, *in vivo* characterization of tissue heterogeneity offers new insights into underlying biology. Instead of increasing spatial resolution to microscopic levels, DWI probes microstructure by analyzing diffusion behavior at the existing voxel resolution. The conventional mono-exponential model is insufficient for assessing tumor heterogeneity due to its reliance on Gaussian water diffusion, which is not typically observed in tumors. Advanced models, also known as signal representation techniques (Novikov *et al* 2018), can capture non-Gaussian diffusion, which reflects tissue irregularity and heterogeneity. This section will focus on two such models, DKI (Jensen *et al* 2005) and FROC (Zhou *et al* 2010) models, while other approaches, such as diffusional variance decomposition (DIVIDE) model (Szczepankiewicz *et al* 2016), will be discussed later.

The DKI model extends the mono-exponential model to account for non-Gaussian diffusion behavior by incorporating a second-order term. As an extension of diffusion tensor imaging, DKI provides two sets of parameters: the diffusion coefficients expressed as a second-rank tensor *D* (in mm^2^ s^−1^) and diffusional kurtosis tensor *K* (dimensionless), a normalized variance metric used as an indicator of tissue heterogeneity.

\begin{equation*}S = {S_0}{\mathrm{exp}}\left[ { - bD + \frac{1}{6}K{{\left( {bD} \right)}^2}} \right].\end{equation*}

The DKI model, an extension of diffusion tensor imaging, has been widely used to study diffusion anisotropy by acquiring images with multiple *b*-values and diffusion gradient directions. In cancer imaging, DKI is often simplified to trace-weighted images to reduce time and minimize sensitivity to diffusion anisotropy. Since its introduction, DKI has been applied to various oncological settings, including cancer detection, diagnosis, tumor grading, staging, classification, and treatment response evaluation (Tang and Zhou 2019). Although most studies focus on brain tumors (Abdalla *et al* 2020), DKI has also been used in research on prostate, breast, lung, kidney, liver, bladder, and ovarian cancers (Rosenkrantz *et al* 2015). Prostate cancer is especially notable, as the recommendation for using high *b*-values (typically ⩾1400 s mm^−2^) is explicitly incorporated into the imaging criteria of prostate imaging reporting and data system (PI-RADS) v2 1 (Turkbey *et al* 2019).

The FROC model is a specific case of continuous-time random walk model (Karaman *et al* 2016), which accounts for intra-voxel diffusion heterogeneity in both time and space. The FROC model simplifies the spatiotemporal characteristics by focusing exclusively on spatial heterogeneity. For a Stejskal–Tanner diffusion gradient *G*_d_ with a duration *δ* and gradient separation Δ, the diffusion-induced signal loss is expressed as:

\begin{equation*}S = {S_0}{\mathrm{exp}}\left[ { - D{\mu ^{2\left( {\beta - 1} \right)}}{{\left(\gamma {G_{\mathrm{d}}}\delta \right)}^{2\beta }}\left( {\Delta - \frac{{2\beta - 1}}{{2\beta + 1}}\delta } \right)} \right],\end{equation*}

where *μ* is a spatial constant, *D* is diffusion coefficient (in mm^2^ s^−1^), and *β* (dimensionless; $0 &lt; \beta \unicode{x2A7D} 1$) originates from a fractional order differential equation and has been correlated with spatial diffusion heterogeneity (Zhou *et al* 2010), decreasing as tissue heterogeneity increases.

In the past decade, intra-voxel heterogeneity has been extensively studied by investigating the relationship between tissue structural heterogeneity and the spatial diffusion heterogeneity parameter, *β*, within the FROC model (Zhou *et al* 2010). Studies using a maximal *b*-value around 4000 s mm^−2^ have revealed significant differences in *β* between pediatric and normal brain tissues, low- and high-grade pediatric brain tumors, and low- and high-grade gliomas in adults (Tang and Zhou 2019). The FROC model’s application in assessing tumor heterogeneity extends beyond brain tumors, with growing interest in its use for breast, prostate, gastric, cervical, endometrial cancers, and other malignancies (Tang and Zhou 2019). While this section primarily focuses on DKI and FROC models due to their demonstrated utility in oncologic applications, it is worth noting that other advanced diffusion models, such as NODDI (Zhang *et al* 2012), CHARMED (Assaf and Basser 2005), and related neuroimaging frameworks, offer complementary perspectives on tissue microstructure and heterogeneity. Comparative evaluation across models originating from both neural and oncologic imaging domains may help identify shared strengths and domain-specific limitations.

##### Current and future challenges

*DWI acquisition*

DKI and FROC models typically require high *b*-values, with DWI acquisition often relying on single-shot EPI due to its time efficiency. While higher *b*-values enhance diffusion contrast and sensitivity, they also reduce SNR due to increased diffusion-induced signal loss and *T*_2_-induced attenuation from longer TE. Achieving an optimal balance between SNR and image contrast often necessitates empirical fine-tuning. Adequate SNR is critical for exploring heterogeneity with high *b*-values; insufficient SNR can compromise the reliability of parametric maps, leading to erroneous tissue characterization. Moreover, diffusion time, which governs the spatial scale of tissue heterogeneity, is usually fixed on commercial scanners. In spin-echo sequences, the shortest diffusion time is limited by gradient strength, while the longest is constrained by the maximum allowable TE to maintain adequate SNR. Additionally, the strong diffusion gradients for high *b*-values can induce vibrations and eddy currents, both of which lead to artifacts. In body imaging, such as breast imaging, additional artifacts can be produced due to off-center positioning, insufficient fat suppression, and gradient non-linearity. Longer acquisition times (e.g. up to 7–10 min for whole-organ DKI and FROC protocols at 3 T (Steven *et al* 2014, Karaman *et al* 2021), required by the increased number of *b*-values and/or the need for signal averaging, further challenge the feasibility of these models. Maintaining an adequate SNR is also critical, as reduced SNR (e.g. <3) at high *b*-values can bias kurtosis and fractional-order parameters.

*Standardization*

A key challenge in advanced DWI, including DKI and FROC models, particularly in advanced models, is the lack of standardized protocols. Variations in acquisition parameters, particularly *b*-value selection, and differences in post-processing can lead to inconsistencies in parameter estimates. While there is relatively more consensus in the conventional mono-exponential model, the issue becomes more pronounced in models that require multiple and/or high *b*-values to characterize non-Gaussian diffusion behavior (Rosenkrantz *et al* 2015). For example, in DKI and FROC, differences in *b*-value distribution and range can impact the estimation of parameters such as DKI’s *K* and FROC’s *β*. In DKI, while at least three *b*-values are required to estimate *K*, in practice, it is recommended to use more than three *b*-values, with at least two above or below 1000 s mm^−2^, for reliable estimates of *D* and *K* (Rosenkrantz *et al* 2015). In DKI, the valid *b*-value range is typically capped at 3000 s mm^−2^, as the quadratic model becomes unstable beyond this point. Studies report significant variability in *b*-value selection, ranging from 3 to 6 *b*-values (0–3000 s mm^−2^) for DKI and 5–12 *b*-values (0–4000 s mm^−2^) for FROC, with some DKI studies extending the maximum *b*-value to 6000 s mm^−2^ (Rosenkrantz *et al* 2015).

Variations in *b*-value selection can lead to considerable discrepancies in parameter estimates across studies. Advanced models such as DKI and FROC involve non-linear fitting algorithms that are sensitive to the number and distribution of *b*-values as well as to SNR. The increased complexity of the models and reduced SNR in high *b*-value images often cause convergence failures, unstable fits, or biologically implausible values. Each model also has inherent limitations: DKI can exhibit systematic errors because it only incorporates terms up to the second order of the cumulant expansion (Jensen and Helpern 2010), while parameter fitting in FROC model can be complicated due to the strong coupling between *D* and *μ* (Zhou *et al* 2010). These challenges underscore the need for effective and transparent fitting strategies applicable across platforms. It is important to note that the efforts in harmonization must be built upon diffusion model validation, especially in the context of their biophysical underpinnings. In practice, standardization and model refinement should progress together, with harmonization efforts providing feedback to improve model robustness and accuracy. Such an integrative approach ensures that standardization enhances both reproducibility and confidence in model validity.

*Interpretability and validation*

The tissue heterogeneity-related parameters, *K* and *β*, have been shown to differentiate different grade of tumors (Tang and Zhou 2019), with higher malignancy associated with increased tissue heterogeneity. Although studies have explored the biophysical basis of *K* and *β*, a consistent relationship between them and the underlying microstructural heterogeneity remains to be established (Maier *et al* 2010). A significant gap persists in the validation of these models against histopathological standards, largely due to the lack of robust quantitative measures for histopathological heterogeneity and the differing spatial scales between DWI-derived metrics and histologic measures. Further work is required to better understand the complex diffusion behaviours as described by the diffusion models and to establish a plausible biological and structural significance of DWI-derived tissue heterogeneity.

##### Advances in science and technology to meet challenges

*DWI acquisition*

Advancements in DWI acquisitions have improved the efficiency of high-*b*-value imaging. Techniques such as high receiver bandwidth, parallel imaging, multi-shot sequences, and/or non-EPI-based acquisitions help reduce susceptibility distortions, while pulse sequences like twice-refocused spin echo (TRSE) (Reese *et al* 2003, Karaman and Zhou 2018), and others methods mitigate eddy current-induced distortions. Simultaneous multi-slice imaging enables faster high-*b-*value, multi-slice DWI acquisitions with reduced repetition time (TR) (Barth *et al* 2016). Time-dependent diffusion sequences, already applied in DKI, enable the exploration of tissue properties across various diffusion time regimes (Reynaud 2017). Despite these innovations, single-shot EPI remains the clinical standard, underscoring barriers to integrating advanced techniques into routine practice. On the other hand, AI-based approaches show promise in addressing challenges of long scan times, image distortion, and low SNR (Lundervold and Lundervold 2019). AI-enhanced image reconstruction methods, such as DL-based reconstruction and compressed sensing with AI, reduce scan time while maintaining high image quality (Knoll *et al* 2020). Commercially available tools such as GE’s AIR™ Recon DL, Siemens’ Deep Resolve, and Philips’ SmartSpeed AI exemplify these innovations, which also include denoising strategies to improve SNR, particularly in high-*b*-values. AI has also enhanced multi-band acquisitions (e.g. GE’s HyperSense), enabling faster and more reliable DWI.

Together, these acquisition and reconstruction advancements help overcome key technical barriers that have historically limited the clinical feasibility of advanced diffusion models. By improving data quality and acquisition efficiency, these techniques can stabilize signal behavior across a range of *b*-values, enabling more robust and repeatable model fitting, particularly for models that require multi-high-*b*-values. Correlation analyses in recent technical studies have shown that variability in derived diffusion parameters is often attributable to differences in protocol design, such as *b*-value distribution, highlighting the importance of harmonization (Ljimani *et al* 2020). These improvements lay essential groundwork for multi-site reproducibility, as seen in ongoing efforts toward IVIM model standardization (section 5) (Baltzer *et al* 2020). The clinical utility of advanced models can be further evaluated through multi-center studies assessing repeatability, biological plausibility (e.g. consistency with expected microstructural trends), and added prognostic or predictive value beyond conventional metrics like ADC. While promising, these techniques remain in early stages of clinical integration and will require continued validation in disease-specific and multi-institutional settings to support broader adoption.

*Standardization*

In recent years, there has been an increasing emphasis on the standardization of DWI protocols to improve greater consistency and reliability of diffusion parameters, laying the foundation for more complex models, such as DKI and FROC. As mentioned in section 5, the IVIM model has been central to these efforts, catalyzing broader initiatives in protocol harmonization and cross-site reproducibility (Baltzer *et al* 2020, Ljimani *et al* 2020). As advanced models gain traction, standardizing tissue heterogeneity modeling across centers will require coordinated strategies, including consensus on acquisition protocols, shared and validated fitting algorithms (Garyfallidis *et al* 2014), benchmarking datasets, and agreed-upon parameter definitions. Standardized diffusion phantoms further support these efforts by providing ground truth measurements to assess scanner performance and inter-site variability. However, harmonization alone is not sufficient for full validation; quantitative verification against *ground truth* remains essential. This includes test phantoms, as well as histological and computational modeling approaches that establish the biological and physical validity of diffusion parameters. These complementary strategies, together with multi-center reproducibility studies, will be key to evaluating model accuracy, repeatability, and prognostic value. Machine learning-based fitting algorithms, often pre-trained and readily available, offer additional promise by simplifying post-processing and improving reproducibility. With continued improvements in parameter estimation techniques, the impact of protocol variation may further diminish, supporting the robust and generalizable adoption of advanced DWI models in clinical research and practice.

*Interpretability and validation*

Recent research has emphasized rigorous histological validation and improved experimental designs and models to explore specific tissue microstructures. Most DWI studies use the traditional single diffusion encoding with Stejskal Tanner gradients, where a single pair of pulsed gradients encode diffusion along one direction per signal readout. This conflates three key diffusion features: microscopic anisotropy, orientation dispersion, and isotropic diffusivity heterogeneity, limiting its ability to probe complex tissue microstructures. To overcome this, newer techniques use multi-directional encoding within a single acquisition (Szczepankiewicz *et al* 2021). Double diffusion encoding, for example, uses parallel and orthogonal diffusion encoding as a probe of local pore geometry (Shemesh *et al* 2010), while tensor-valued diffusion encoding uses continuously varied gradient waveforms to optimize encoding, shifting the description from a vector to a b-tensor for more comprehensive diffusion encoding (Szczepankiewicz *et al* 2021). Diffusion encoding with varying b-tensor shapes allows for diverse analytical methods (Westin *et al* 2016). The DIVIDE model, an extension of the traditional DKI, decomposes diffusional variance into two components (Szczepankiewicz *et al* 2016). The diffusional variance due to microscopic anisotropy and isotropic heterogeneity have been linked specifically to cell eccentricity and cell density variance, as confirmed through quantitative microscopy (Szczepankiewicz *et al* 2016). Additionally, microscopic fractional anisotropy reflects diffusion anisotropy without the effects of orientation dispersion, distinguishing tissues with different heterogeneity levels, such as invasive ductal carcinoma from normal fibroglandular breast tissue (Cho *et al* 2022). Voxel-wise ADC and cell density correlations in *ex vivo* human brain samples have also enabled prediction of histological features from MRI measurements (McGarry *et al* 2016). Another approach combines diffusion MRI and histology data in a joint model, using data fusion to investigate white matter diffusion properties when multiple modalities inform the ‘true’ tissue microstructure (Howard *et al* 2019). While not directly targeting tissue heterogeneity, they can complement DKI, FROC, DIVIDE, and other models. Advances in AI-driven digital pathology further enhance histology-MRI correlations through automated analysis (Deng *et al* 2020).

##### Concluding remarks

Using DKI and FROC models as examples, we have shown that advanced diffusion imaging has emerged as a valuable tool for probing tissue heterogeneity, offering additional insights into tumor microstructure. Despite the significant progress in acquisition strategies, model development, and the incorporation of AI technologies, several challenges remain. The challenges to balance an adequate SNR at high *b*-values, the need for standardized protocols, and the interpretability of model parameters lead to active areas of ongoing research. Recent advancements, including the development of more efficient acquisition techniques, AI-enhanced image reconstruction and processing, and the increasing integration of histological information, offer promising solutions to these challenges. As the field moves forward, continued collaboration across research centers and the standardization of imaging protocols and fitting strategies will be key to unlocking the full potential of diffusion models for tissue heterogeneity studies. Ultimately, these advancements will enhance our ability to noninvasively characterize tumor heterogeneity, improving diagnosis and monitoring of RT.

### Acknowledgements

This work was supported in part by the NIH Grants R21EB032071, R01EB026716, and 1S10RR028898.

### 7. DWI for cell size imaging

#### Junzhong Xu, John C Gore, and Xiaoyu Jiang

Institute of Imaging Science, Vanderbilt University Medical Center, Nashville TN, United States of America

##### Status

DWI is an imaging technique that allows for the non-invasive probing of biological tissue microstructure without exogenous agents. Due to its high sensitivity to morphological variations at the cellular level, DWI has become a widely utilized method for detecting disease progression and assessing responses to therapeutic interventions. Early studies have demonstrated an inverse correlation between DWI-derived ADC and histology-derived tumor cellularity (Sugahara *et al* 1999), suggesting ADC could be a surrogate imaging biomarker for cellularity. This is particularly significant in oncology, where many effective anti-cancer treatments lead to a marked reduction in cell density *before* observable changes in tumor volume—the current imaging standard for evaluating treatment response. Consequently, ADC has emerged as a promising imaging biomarker in oncology for assessing tumor cellularity. However, ADC does not always correlate with cellularity (Surov *et al* 2017) because it reflects averaged diffusion properties influenced by various concurrent tissue characteristics e.g. both cellularity and cell size, which may compete and diminish the sensitivity to cellularity.

Advanced multi-compartment diffusion methods have been developed to quantitatively characterize tissue properties to address this limitation. These DWI techniques typically involve data acquisition using multiple diffusion times and *b*-values, coupled with a two-compartment biophysical model to distinguish intra- and extra-cellular spaces. Through this approach, tissue microstructural properties such as mean cell size, intracellular volume fraction, and diffusivities can be non-invasively extracted. Since only mean cell size can be reliably estimated (as discussed below), this approach is termed MR cell size imaging (Jiang *et al* 2021), with representative methods including VERDICT (vascular, extracellular and restricted diffusion for cytometry in tumors) (Panagiotaki *et al* 2014), IMPULSED (imaging microstructural parameters using limited spectrally edited diffusion) (Jiang *et al* 2016), and POMACE (pulsed and oscillating gradient MRI for assessment of cell size and extracellular space) (Reynaud *et al* 2016). Note that, although underestimated, cellularity derived from these methods shows a good correlation with histologic ground truths, suggesting it still can be used as an indicator to report cellularity or cell density in tumors.

To date, the VERDICT and IMPULSED methods have progressed from preclinical validation to clinical imaging research across multiple cancer types, demonstrating promising potential for clinical application in cancer management (Johnston *et al* 2019, Wu *et al* 2022). These two methods assume a two-compartment (intra- and extra-cellular spaces) model and hence diffusion-weighted MRI signal can be written as

\begin{equation*}S\left( {b{,_{{\Delta_\mathrm{diff}}}}} \right) = {v_{{\mathrm{in}}}}{S_{{\mathrm{in}}}}\left( {d,{D_{{\mathrm{in}}}}|b,{{ }_{\Delta_{\mathrm{diff}}}}} \right) + \left( {1 - {v_{{\mathrm{in}}}}} \right){S_{{\mathrm{ex}}}}\left( {{D_{{\mathrm{ex}}}},{ }{\beta _{{\mathrm{ex}}}}|b,{{ }_{\Delta_{\mathrm{diff}}}}} \right),\end{equation*}

where ${\Delta _{{\mathrm{diff}}}}$ is the effective diffusion time, ${S_{{\mathrm{in}}}}$ and ${S_{{\mathrm{ex}}}}$ are intra- and extracellular dMRI signals, respectively, $d$ is the mean cell size, ${D_{{\mathrm{in}}}}$ is intracellular diffusivity, ${D_{{\mathrm{ex}}}}$ is the extracellular diffusivity, and ${\beta _{{\mathrm{ex}}}}$ is the extracellular diffusivity dispersion rate that is used in the IMPULSED method. The analytical expressions of ${S_{{\mathrm{in}}}}$ and ${S_{{\mathrm{ex}}}}$ have been reported for VERDICT (Panagiotaki *et al* 2014) and IMPULSED (Jiang *et al* 2016, Xu *et al* 2020) methods, respectively. A major distinction between VERDICT and IMPULSED methods is that IMPULSED incorporates data acquisition using both the conventional pulse gradient spin echo (PGSE) sequence and the oscillating gradient spin echo (OGSE) sequence, while VERDICT relies solely on PGSE acquisitions. Because OGSE probes much shorter diffusion times than PGSE, IMPULSED encompasses a broader range of diffusion times, enhancing sensitivity to typical cancer cell sizes (e.g. 10–20 *μ*m) and intracellular diffusivities. The IMPULSED method has undergone comprehensive validation through computer simulations, *in vitro* studies with cultured cells, and *in vivo* studies with animal models, supported by histological analysis (Xu *et al* 2020. These validations confirm that cellular-level microstructural information, such as mean cell size, can be reliably estimated *in vivo* in cancer patients using the IMPULSED method (Xu *et al* 2020).

A series of preclinical studies have demonstrated that various anti-cancer treatments induce significant cellular morphological changes, particularly in cell size, across different tumor types and treatments such as chemotherapy, radiotherapy, and immunotherapy (Jiang *et al* 2021). Notably, these cellular changes typically occur well before observable alterations in tumor volume. For instance, cell shrinkage is a hallmark of apoptosis, making imaging of cell size a valuable tool for detecting treatment-induced apoptosis. This, in turn, offers a novel approach for characterizing early therapeutic response and predicting treatment efficacy. Therefore, there is growing interest in implementing MR cell size imaging in clinical MRI scanners for cancer patients. Proof-of-concept studies have shown that IMPULSED-derived cell size is associated with Gleason scores in prostate cancer (Wu *et al* 2022) and can be used to classify low- and high-grade gliomas, as well as molecular subtypes in breast cancer. These findings open new avenues for utilizing MRI-derived cell size and cellularity to assess tumor status and treatment response at the cellular level.

##### Current and future challenges

Despite the success of MR cell size imaging in both preclinical and clinical applications, several challenges remain that prevent these methods from becoming standard-of-care imaging.

*Challenges in modeling*

MR cell size imaging relies on a two-compartment model (intra- and extra-cellular spaces) that overlooks two tissue features: (i) inhomogeneous *T*_2_ relaxation and (ii) transcytolemmal water exchange. Studies have shown that the latter leads to a significant underestimation of the intracellular volume fraction, though its impact on mean cell size estimation is negligible (Li *et al* 2017). Consequently, cell size is the only tissue property that can be accurately fitted, which is why the technique is referred to as MR cell size imaging. Although cell density and/or cellularity are significantly underestimated, the fitted values still correlate strongly with ground-truth values, suggesting that an indicator of cell density can be derived from MR cell size imaging. Nevertheless, incorporating both *T*_2_ relaxation and transcytolemmal water exchange into the biophysical model would allow for more comprehensive tissue property extraction (Jiang *et al* 2022). Another modeling challenge is related to tumor microstructure. MR cell size imaging has been successfully implemented in breast, prostate, and brain cancers. However, its oversimplified two-compartment model may not be suitable for some tumors with a complex mixture of different tissue types, such as in the glioblastoma lesions where cancer cells infiltrate into the white matter, resulting in multiple compartments including cells, axons, and extracellular space. A more comprehensive biophysical model is plausible under such circumstances.

*Challenges in acquisition*

As a DWI-based method, MR cell size imaging inherits both the strengths and weaknesses of DWI. The IMPULSED method, for instance, utilizes OGSE sequences, which are not standard pulse sequences but are available as work-in-progress or research patches from major vendors. This limits the routine clinical use of OGSE acquisitions. Additionally, the use of OGSE sequences in IMPULSED for shorter diffusion times and the use of higher *b*-values in VERDICT for stronger diffusion weighting often result in longer TEs, which can reduce image quality and SNR (Xu 2021). Recent clinical studies have demonstrated that IMPULSED was successfully implemented with single-axis gradient strengths of 45 mT m^−1^ (Wu *et al* 2022) and 65 mT m^−1^ (Xu *et al* 2020), indicating its broader clinical applicability. However, typical MR-LINAC systems usually have lower field strengths (e.g. 1.5 T or 0.35 T) and relatively lower gradient strengths (e.g. 30 mT m^−1^), both could further reduce SNR and longer diffusion times.

*Challenges in data analysis*

MR cell size imaging depends on fitting complex biophysical models to derive tissue microstructural information. Analytical equations are typically required to link microstructural and pulse sequence parameters to DWI signals, but these equations depend on realistic gradient waveforms and require significant support from medical physicists to calibrate them (Xu 2021), such as using the ice-water phantom (Malyarenko *et al* 2013). Furthermore, the relatively lower SNRs in MR cell size imaging make data fitting more challenging, and advanced fitting methods often require high computing resources and extended running times, which are not ideal for clinical settings (Xu *et al* 2025). Currently, data analysis for MR cell size imaging is usually performed off-scanner, so developing a fast, user-friendly data analysis routine that can be executed directly on clinical MRI scanners is a critical need.

##### Advances in science and technology to meet challenges

Significant advances have been made in further development and optimization of MR cell size imaging to address the challenges mentioned above.

*Advances in modeling*

Integrating different intra- and extra-cellular *T*_2_ relaxation times into biophysical models for MR cell size and cellularity imaging is relatively straightforward. However, this approach requires longer scan times due to the need for multiple TEs in acquisition. Despite this, *T*_2_ relaxation has already been incorporated into both the VERDICT and IMPULSED methods (Palombo *et al* 2023), leading to more accurate estimations of cellularity and cell size. In contrast, incorporating transcytolemmal water exchange into models is more challenging due to the complex interplay between water diffusion and exchange during the finite duration of diffusion gradients. Recent advances have simplified this issue by disregarding the effects of water exchange on OGSE acquisitions due to short diffusion times and correcting for the exchange effects on PGSE acquisitions, both of which result in improved estimates of both cell size and cellularity (Jiang *et al* 2022). Nonetheless, more accurate biophysical models are needed to fully address this challenge.

*Advances in acquisition*

Accelerating data acquisition while maintaining sufficient SNR is crucial for MR cell size imaging. As a subclass of DWI, MR cell size and cellularity imaging can benefit from rapid advancements in DWI acquisition techniques such as multi-band imaging and compressed sensing. Another promising approach is to optimize MR cell size imaging protocols using methods like the Cramér–Rao lower bound to minimize total scan time while maintaining reasonable precision (Brihuega-Moreno *et al* 2003). It is important to note that optimized acquisition protocols may vary across different MRI scanners due to differences in hardware specifications, necessitating the involvement of physicists to tailor protocols to specific systems.

*Advances in data analysis*

MR cell size imaging can also benefit from recent advancements in data fitting techniques. Traditional non-linear least squares fitting methods are vulnerable to the relatively low SNRs on clinical MRI scanners, which can reduce fitting robustness. Advanced techniques such as denoising with PCA (Veraart *et al* 2016b) or DL, and improving fitting accuracy and robustness through Bayesian approaches, dictionary matching, or machine learning, offer promising solutions (Xu *et al* 2025). Additionally, developing a user-friendly, click-button software package for advanced DWI data fitting that is integrated into MRI scanner consoles, would greatly reduce the barriers for clinicians in adopting MR cell size and cellularity imaging in clinical practice (Xu *et al* 2025). Collaboration with vendors would be necessary to achieve this level of integration.

##### Concluding remarks

The recent development of DWI-based MR cell size and cellularity imaging offers a novel approach to extracting microstructural information at the cellular level. Extensive preclinical validations and promising proof-of-concept studies in cancer patients have demonstrated its potential in characterizing tumor status and monitoring early therapeutic responses. This innovation paves the way for using MRI-derived cell size and cellularity as surrogate imaging biomarkers to provide a more comprehensive characterization of tumors at the cellular level.

### Acknowledgments

This work was funded by NIH Grants R01 CA269620, R01 DK135950, and R21 CA270731.

### Concluding statement for chapter II

*Sirisha Tadimalla*

Institute of Medical Physics, The University of Sydney, Sydney, Australia

The traditional ADC model has long provided a simple, fast and accessible measure of overall diffusion and is widely used in RT. However, its reliance on a single compartment, Gaussian diffusion assumption imposes limitations on its ability to resolve the complex, multi-compartmental microenvironments found in tumors. The advanced diffusion models described in this chapter aim to address these limitations by capturing specific tissue microstructural features such as microcirculation, heterogeneity and cell size. A common theme across these methods is the potential to detect early biological changes in tumors, offering a more nuanced view of treatment response than size-based measures. Some models, like RSI and IVIM, have begun to see clinical use in specific cancers, and early applications in RT —such as hypoxia imaging and microstructural response assessment—are emerging, however, their readiness for individual patient analysis varies. Furthermore, most models rely on simplified biophysical assumptions, and many derived parameters lack direct histological validation, making their interpretation difficult on a per-patient basis. As we move toward incorporating biological information from DWI into RT planning, adaptation, and response monitoring, validation against histopathological standards, refinement of model interpretability, technical optimization, and the development of consensus standards will be essential to ensure these new tools are both scientifically robust and clinically feasible.

## Chapter III. Clinical perspectives on DWI applications in radiation oncology

### 8. The role of DWI in brain tumor RT

#### Jill B De Vis

Department of Radiation Oncology, Harold C. Simmons Comprehensive Cancer Center, University of Texas Southwestern Medical Center, TX, United States of America

Advanced Imaging Research Center, University of Texas Southwestern Medical Center, Dallas, TX, United States of America

##### Status

DWI allows for the evaluation of physiologic properties that cannot be identified by conventional MR imaging sequences. DWI measures may relate to histopathological markers of cellular density and proliferation (Barajas *et al* 2013). DWI may identify tumor invasion beyond traditional MRI defined margins (Bobholz *et al* 2022), and can differentiate infiltrative edema from vasogenic edema in glioblastoma multiforme (GBM) patients (Figini *et al* 2023). In addition, by virtue of its property identifying hypercellular areas, it may also depict cellularity-related tissue hypoxia (Barajas *et al* 2013, Hino-Shishikura *et al* 2014), which is an important biomarker of radiation resistance. The above properties make DWI an important imaging sequence to consider when implementing BIgART.

*DWI may predict tumor outcome*

Numerous studies have investigated the value of DWI as a prognosticator for intracranial tumor outcome. In GBM patients, most but not all studies have reported a positive association between DWI parameters and patient’s outcome (Brancato *et al* 2020). Additionally, there are some reports that identified the volume of DWI-detected hypercellular area to be a more adverse prognostic marker than the gross tumor volume as identified on post-contrast *T*_1_-weighted imaging (Lawrence *et al* 2023). Other than DWI’s utility in predicting GBM patient’s outcomes at the start of treatment, radiation-induced changes in DWI and its associated measures have predictive value. More pronounced increases in ADC values throughout radiation treatments, or the lack thereof, have been shown to be predictive of progression-free survival and/or overall survival. Similar to DWI’s utility in predicting outcome in GBM patients, increases in ADC during and after treatment have also been shown to be predictive of treatment success irrespective of changes in tumor volume (Chuang *et al* 2012).

*DWI may predict tumor recurrence location*

Multiple groups have evaluated the spatial relationship between DWI abnormalities and location of tumor recurrence. Mostly, low-ADC regions at diagnosis were shown to be at risk for future tumor recurrence even when they are located within area that is hyperintense on *T*_2_-weighted imaging in GBM patients (Khalifa *et al* 2016, Zhang *et al* 2019). Further proof that DWI techniques may be able to identify hypercellular areas or tumor infiltration outside the gross tumor volume, comes from a DTI or tractography study that validated tumor infiltration as identified on qualitative assessment of anisotropic (*q*) value maps through a comparison with multivoxel proton magnetic resonance spectroscopy (Yan *et al* 2019).

*DWI may differentiate pseudoprogression or radiation necrosis from tumor progression*

A common conundrum that is encountered when treating intracranial malignant disease, is the differentiation between radiation necrosis and tumor progression. Multiple imaging sequences, including DWI, have been evaluated for their accuracy in distinguishing both entities. ADC was found to have a slight superior performance over perfusion-weighted imaging (PWI) in differentiating pseudoprogression from tumor progression with a sensitivity and specificity of 88% and 85%, respectively, compared with the respective values of PWI, 85% and 79% (Tsakiris *et al* 2020). DKI, specifically relative mean kurtosis performed similar to dynamic susceptibility contrast-enhanced relative cerebral blood volume assessment, both with a diagnostic accuracy of 82% (Shi *et al* 2021). Data on the selective size imaging using filters via diffusion times (Devan *et al* 2022) is more limited but is promising.

##### Current and future challenges

*Efficacy of DWI-guided radiation treatment*

A multitude of studies in GBM and high-grade glioma patients has demonstrated an association between DWI and clinical outcome/areas of tumor recurrence. Despite this, the impact of DWI-guided RT on patients’ outcomes has not been investigated. A small-scale retrospective study did report on conventionally planned radiation isodose line volumes and their overlap with DWI-hypercellular volume. In this study, it was found that 67% of GBM patients had incomplete coverage of their DWI-hypercellular volume by the 95% isodose line, and that this was a significant predictor for progression free survival (Pramanik *et al* 2015). The latter underscores the significance of DWI assessment in high grade glioma RT planning, and its potential to improve the therapeutic efficacy of radiation in those patients. Other than DWI’s potential in high-grade glioma patients, studies have also assessed the influence of DWI assessment on brain metastases radiosurgery treatment planning, and its relationship with areas of tumor recurrence. This analysis found that DWI-guided radiosurgery delineation would have covered a greater volume of subsequent tumor recurrence than conventional radiosurgery planning (Zakaria *et al* 2017). Despite these promising findings, the direct impact of DWI-guided treatment on brain tumor patient outcomes remains unexplored. Addressing this gap will require the development of robust DWI-guided treatment planning strategies that integrate both tumor delineation and studies evaluating appropriate prescription doses.

*DWI sequence optimization and standardization*

Variation in acquisition parameters presents a significant barrier to the generalization and clinical implementation of DWI-guided RT. Threshold values reported in the literature may not be directly applicable across institutions due to differences in scanner hardware, acquisition protocol, and reconstruction methods. To ensure reliable and reproducible use of DWI in RT planning, it is essential to standardize pulse sequence parameters and post-processing algorithms. Such standardization would facilitate multi-center adoption and support the accurate integration of DWI into clinical workflows.

##### Advances in science and technology to meet challenges

*Implementation of DWI-adaptive radiation treatments*

MR-LINAC systems are novel treatment devices that allow for daily online adaptive RT. The 1.5 T MR-LINAC system supplies a DWI sequence shown to accurately measure ADC within the clinical target volume (CTV) when compared to a diagnostic MRI scanner (Lawrence *et al* 2021), has good repeatability (Kooreman *et al* 2019), and is able to identify longitudinal changes in ADC values over time (Lawrence *et al* 2023). Thus far, MR-LINACs are mainly used to adapt treatment volumes to anatomical changes detected by conventional MRI sequences (Maziero *et al* 2021). Theoretically, daily DWI evaluation on MR-LINAC could allow for dose de-escalation and/or volume reduction when hypercellularity resolves during the radiation course, or dose- and volume-escalation if new areas of hypercellularity, i.e. progression, occurs.

*DWI assessment*

To enable the clinical implementation of DWI-guided RT, strategies need to be developed to support the reliable and reproducible detection and delineation of DWI abnormalities. It is well recognized that traditional manual segmentation is labor-intensive and highly dependent on the experience of radiation oncologists, who must integrate information from multiple MRI contrasts (e.g. post-contrast *T*_1_-weighted, fluid-attenuated inversion recovery (FLAIR)) to delineate tumor, edema, resection cavity, gross tumor volume (GTV), and CTV. Prior studies have demonstrated the feasibility of supervised DL models for daily MRI segmentation on MR-LINAC systems, and others have incorporated multimodal MRI priors to automatically segment GBM (Breto *et al* 2023, Tian *et al* 2024). Physicians often use threshold-based approaches on ADC maps because ADC correlates with cellularity, is relatively robust, and can be rapidly interpreted. However, thresholding cannot reliably distinguish tumor from non-tumor tissues: ADC values overlap substantially with normal white matter, treatment-related effects, and heterogeneous infiltrative tumor components. There is no universally accepted ADC threshold that defines tumor burden, particularly in the complex microenvironment of the brain. To address this unsolved issue, DL framework should incorporate supervised training using multimodal MRI, not ADC alone. Also, leveraging physician-defined contours based on the full MRI set (e.g. *T*_1_-weighted, post-contrast *T*_1_-weighted, FLAIR) together with DWI/ADC, and linking these to clinical outcomes, such as patterns of recurrence and radio-resistant subregions. By anchoring DL training to outcome-relevant ground truth rather than thresholding values, the model can learn to identify tumor subregions that are associated with poor response.

##### Concluding remarks

DWI holds a promising spot in improving the efficacy of RT of intracranial malignant disease. However, it is not the only imaging technique that holds promise in identifying areas at risk for disease recurrence. The main challenge in BIgART is to assess the added value of each individual imaging sequence, and to weigh this value against the time-burden that it imposes on the patient. Similarly, it remains to be elucidated whether BIgART using a single time point MRI scan suffices, or if more datapoints are needed to adapt the treatment as a response to radiation efficacy.

### 9. The role of DWI in head and neck cancer (HNC) RT

#### Lise Wei

Department of Radiation Oncology, University of Michigan, Ann Arbor, MI, United States of America

##### Status

DWI has become a standard diagnostic tool for HNC due to its compatibility with standard MRI systems, and lack of contrast agent requirement. Its primary uses include tissue characterization, nodal staging, therapy monitoring, and differentiation between recurrent tumors and post-therapeutic changes. It also aids in distinguishing viable from necrotic tumor regions and in evaluating salivary and thyroid gland function (Thoeny *et al* 2012).

*DWI can predict tumor outcome*

DWI demonstrates considerable promise for predicting therapeutic outcomes in patients undergoing chemotherapy or RT for HNC. Lesions with a larger low ADC volume pre-RT have been linked to better outcomes in locally advanced HNSCC (Cao *et al* 2023), though this prognostic value may depend on human papillomavirus status, with differential ADC outcomes observed in p16+ and p16− tumors (Cao *et al* 2019). Another study found similar results that pre-treatment ADC value of complete responders was significantly lower (*p* < 0.05) than that from partial responders (Kim *et al* 2009). Moreover, changes in ADC values during therapy have predictive relevance; specifically, patients who achieve post-treatment complete response (CR) exhibit a significant mid-treatment increase in mean ADC compared to baseline, whereas non-CR patients show minimal or no significant ADC change (Head *et al* 2023). Recent clinical trials highlight the utility of DWI in adaptive RT, using ADC and blood volume maps to guide radiation boosts, which has shown reduced locoregional failure rates (Mierzwa *et al* 2022).

*DWI can differentiate radiation necrosis from tumor progression*

In post-treatment settings, DWI’s ability to distinguish residual or recurrent cancer from benign treatment changes such as radiation necrosis further enhances its clinical relevance. In one meta-analysis, the overall mean ADC values of recurrent lesions was 1.03 × 10^−3^ mm^2^ s^−1^ and that of the posttreatment changes was 1.51 × 10^−3^ mm^2^ s^−1^ (Baba *et al* 2022). The ADC value of recurrence was significantly less than that of posttreatment changes in HNC. The threshold of ADC values between recurrent lesions and posttreatment changes was suggested to be 1.10 × 10^−3^ mm^2^ s^−1^. Another study suggested that the ADC ratio calculated from ADC_1000_ and ADC_2000_ has better differentiation power (Hwang *et al* 2013). When combined with conventional morphological MRI, DWI improves diagnostic accuracy, leveraging the difference in *T*_2_-weighted signal intensities—typically higher in residual HNC compared to fibrosis, despite similar low ADC values (Ailianou *et al* 2018).

*DWI can evaluate salivary gland and swallowing function*

Xerostomia is a late toxicity of concern as it constitutes a significant quality-of-life burden and is often unavoidable due to the involvement of salivary glands in therapy volumes. DWI has recently been used for evaluating post-RT salivary gland function and xerostomia severity (Ermongkonchai *et al* 2023). A study by Loimu *et al* conducted post-treatment ADC analyses of the parotid glands of HNC patients treated with IMRT, and found higher maximum ADC values during stimulation compared to pre-irradiation (*p* < 0.001) (Loimu *et al* 2017). Additionally, a post-RT ADC analyses by Zhang *et al* on nasopharyngeal cancer patients found that those receiving lower radiation doses to the salivary glands had lower maximum ADC measurements during gustatory stimulation and thus had higher likelihood of functional recovery (Zhang *et al* 2018). These findings underscore DWI’s potential role in monitoring and potentially mitigating radiation-induced toxicities by optimizing treatment planning and follow-up strategies.

##### Current and future challenges

A key challenge in utilizing DWI for HNC lies in the variability of ADC thresholds for different tasks, such as tissue characterization and nodal staging. ADC values are influenced not only by tissue properties but also by the diverse methods of ADC calculation, variations in *b*-values, diffusion times, and pulse sequences. This inconsistency complicates the use of ADC as a reliable clinical biomarker, as it is not a specific measure of cancer microstructure. Factors such as cellularity, cell size, nuclear size, and membrane permeability further contribute to ADC variability, limiting its specificity in clinical settings (Jiang *et al* 2016).

Another significant challenges in many of these studies is the small sample size, which limits the reliability of the results. Small sample sizes increase the risk of overfitting models and reduce the statistical power of the findings, making it difficult to generalize the results. Therefore, before implementing these findings in clinical practice, they need to be validated in larger, more diverse patient populations. Expanding study cohorts and conducting multicenter trials will be essential to confirming the reproducibility and robustness of the emerging DWI-based biomarkers.

Additionally, the effects of cancer therapies on tissue, such as early responses of increased membrane permeability, cell swelling, edema, and late effects of cell death, necrosis, and fibrosis, have conflicting influences on ADC values. These variations can reduce the utility of ADC as a reliable biomarker for therapy response (Cao *et al* 2024). False positives, particularly in differentiating residual or recurrent disease from post-RT changes like fibrosis and scar tissue, remain a significant issue (Verma *et al* 2013). Although majority of studies have reported lower ADC values in tumor recurrence than in treatment necrosis, one study reported significantly higher ADC in tumor recurrence than in treatment necrosis, which could be due to greater extracellular space or necrotic regions within high-grade tumors (Sundgren *et al* 2006). The ADC values in treatment necrosis can also be lower because of scarring (Lu *et al* 2004). The effects of infiltration and proliferation have also been noted to confound the straightforward interpretation of ADC value as well (Price *et al* 2004).

Moreover, the head and neck region presents unique challenges due to its complex anatomy, comprising heterogeneous tissues such as fat, muscle, and air, and its geometric irregularities. These factors result in low SNR and susceptibility artifacts, particularly near tissue boundaries and dental fillings. While rapid EPI sequences help mitigate motion artifacts, they are susceptible to geometric distortions and other inherent artifacts such as chemical shift and Nyquist ghosting. Motion artifacts, including those caused by swallowing and breathing, further degrade image quality in this region.

##### Advances in science and technology to meet challenges

As outlined, multiple factors, such as cell membrane permeability changes, apoptosis, necrosis, fibrosis, and scanning parameters, influence ADC values in HNC (Lu *et al* 2004, Sundgren *et al* 2006, Ogura *et al* 2011, Verma *et al* 2013, Li *et al* 2016, Fliedner *et al* 2020). A promising approach to address these limitations is time-dependent diffusion imaging, which enables more precise probing of tissue microstructure and tumor cellular properties. By acquiring multiple diffusion images with varying diffusion times, researchers can better isolate the effects of tissue microstructure on water signals, enabling the quantification of specific tumor cellular parameters. These microstructural parameters, derived from time-dependent diffusion, hold promise as more specific biomarkers than conventional ADC-based metrics. Two primary modeling approaches have emerged to map microstructural features. The first approach models cells as impermeable spheres, confining water molecules in intracellular and extracellular compartments. When diffusion time is much shorter than the residence time of water molecules in the cell, this model proves effective. Techniques such as OGSE have been applied to achieve short diffusion times, with animal studies demonstrating improved detection of hepatocellular carcinoma using this method (More details in section 7). The second approach, the random walk with barriers methodology (RWBM), accounts for the permeable nature of cell membranes. *In vivo* studies using RWBM in oropharyngeal squamous cell carcinoma have shown correlations between tumor volume-to-surface ratio and clinical staging, highlighting the potential clinical relevance of this method (Cao *et al* 2024).

To address imaging artifacts, advanced techniques like PROPELLER (also known as BLADE) have been developed, which acquire data using a modified radial scheme scheme (Pipe 1999). This approach is more resistant to motion artifacts and susceptibility issues, offering improved image quality for the head and neck region. However, it comes with trade-offs, such as longer acquisition times and lower SNR (Li *et al* 2011, Fu *et al* 2021). Despite these limitations, optimizing DWI protocols and exploring new acquisition techniques will be crucial in overcoming these challenges and unlocking DWI’s full potential in HNC treatment (Holdsworth *et al* 2019). More advanced methods have been introduced to overcome these constraints, including TurboPROP (Pipe and Zwart 2006) and its 3D variant radially oriented tri-dimensionally organized readouts (Aboussouan and Pipe 2009), which reduce motion artifacts, scan time, and specific absorption rate (Pipe and Zwart 2006, Tamhane and Arfanakis 2009). DL techniques have also shown promise for mitigating artifacts and generating higher quality images (Fujima *et al* 2023, Kiryu *et al* 2023, Pocepcova *et al* 2025).

##### Concluding remark

DWI has become a standard-of-care imaging technique for HNC diagnosis, especially when combined with other imaging modalities such as *T*_1_/*T*_2_-weighted MRI and CT. Despite its widespread use, its clinical application is still hampered by issues like geometric distortion, low spatial resolution, variability in ADC thresholds, and small study sample sizes. However, new technological advancements are actively addressing these challenges, offering promising potential to enhance diagnostic accuracy and support personalized treatment strategies for HNC patients. As these innovations continue to evolve, DWI could play an even more pivotal role in advancing cancer care. Nonetheless, caution is warranted to adopt standard DW-EPI sequences without adequate quality assurance or repeatability studies, which may impact the reliability of clinical decisions.

### 10. The role of DWI in prostate cancer RT

#### Anna M Dornisch^1^, Christopher C Conlin^2^ and Tyler M Seibert^1,2,3,4^

^1^ Department of Radiation Medicine and Applied Sciences, University of California San Diego Health, La Jolla, CA, United States of America

^2^ Department of Radiology, University of California San Diego Health, La Jolla, CA, United States of America

^3^ Department of Urology, University of California San Diego Health, La Jolla, CA, United States of America

^4^ Department of Bioengineering, University of California San Diego Jacobs School of Engineering, La Jolla, CA, United States of America

##### Status

Multi-parametric MRI (mpMRI) is the current imaging modality of choice for detection and local staging of prostate cancer. Expressly for informing biopsy decisions, the PI-RADS guidelines and subsequent updates have standardized mpMRI to include *T*_2_-weighted MR (*T*_2_W), DWI, and DCE MRI (Turkbey *et al* 2019). However, their prominence in diagnostic radiology influences other clinical uses of mpMRI, such as RT planning.

Two specific applications of mpMRI in prostate RT are: (1) MRI-guided stereotactic body RT (SBRT) and (2) MRI delineation of intraprostatic lesions for focal boost, the application of additional radiation dose to MRI-visible tumor volume (Gao *et al* 2007, McLaughlin *et al* 2010). The MIRAGE trial demonstrated the clinical significance of improved visualization/delineation of the prostate with mpMRI (Kishan *et al* 2023). In MIRAGE, MRI-guided SBRT with a 2 mm planning target volume (PTV) margin was compared to CT-guided SBRT with a 4 mm PTV margin. Significant reductions were observed in both physician-scored toxicity and patient-reported decrement in quality of life in the group with smaller PTV margins, proving that even small changes in target volumes can have meaningful impact on patient outcomes. While *T*_2_W MRI allows for the prostate boundary delineation necessary for SBRT, DWI plays an increasing role in delineation of targets for MRI-guided focal boost (Pathmanathan *et al* 2019).

The focal lesion ablative microboost in prostate cancer (FLAME) trial provides strong evidence that MRI-guided external beam RT focal boost for localized prostate cancer increases biochemical disease-free survival without increasing toxicity (Kerkmeijer *et al* 2021). Evaluation of the contours in the FLAME trial revealed considerably different interpretations of mpMRI in tumor contouring between institutions, but all institutions weighted the ADC map the most (van Schie *et al* 2018).

**Figure 3.**
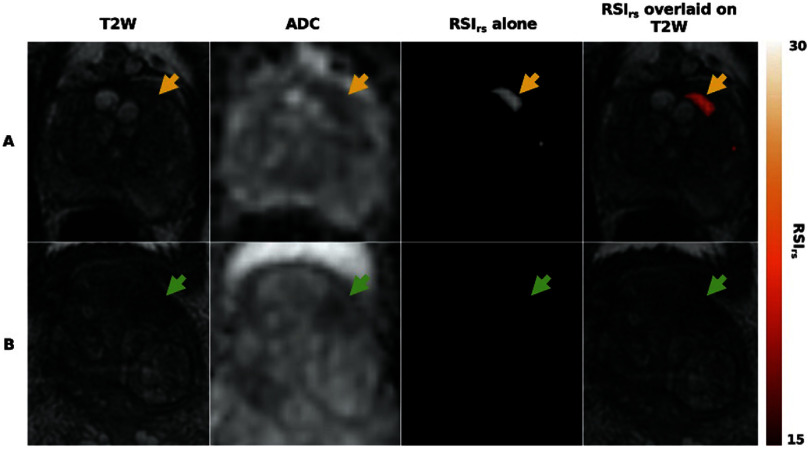
Axial images of *T*_2_W, ADC, and RSIrs. Patient A: prostate imaging-reporting and data system PI-RADS 3 lesion (yellow arrow) in the left transition zone; he underwent prostatectomy and was found to have Gleason 3 + 4 prostate cancer. Patient B: PI-RADS 5 lesion (green arrow) on multiparametric MRI, with subsequent biopsy showing benign prostatic tissue with acute and chronic inflammation. The RSIrs map readily highlights the cancer for patient A. The RSIrs map for patient B has no false-positive voxels (and is shown on the same color scale as the map for patient A). Adapted from Zhong *et al* (2023a). CC BY 4.0.

The ability to identify prostate tumors on MRI is increasingly critical for implementation of focal boost (Dornisch *et al* 2024). Even expert subspecialty radiologists show substantial variability in lesion identification (Anwar *et al* 2014, Steenbergen *et al* 2015). Moving beyond ADC maps, a quantitative diffusion biomarker for prostate cancer, called the RSI restriction score (RSIrs) has been developed that highlights the restricted intracellular diffusion characteristic of prostate cancer. Like other advanced diffusion models, RSI uses additional parameters to separate diffusion signals from various tissues within a single voxel. A four-component model optimally characterizes diffusion within the prostate, with the slowest diffusion compartment yielding the greatest tumor conspicuity (Conlin *et al* 2021). When compared to conventional ADC, RSIrs led to improved voxel-level classification of prostate cancer with lower false positive rate (figure 3) (Feng *et al* 2021). Furthermore, it has been shown to correlate not only with the grade group among whole tumors but also at the voxel-level within individual prostate cancer regions on whole mount histopathology, facilitating radiation focal boost treatment planning (Liss *et al* 2015, Yamin *et al* 2016).

**Figure 4.**
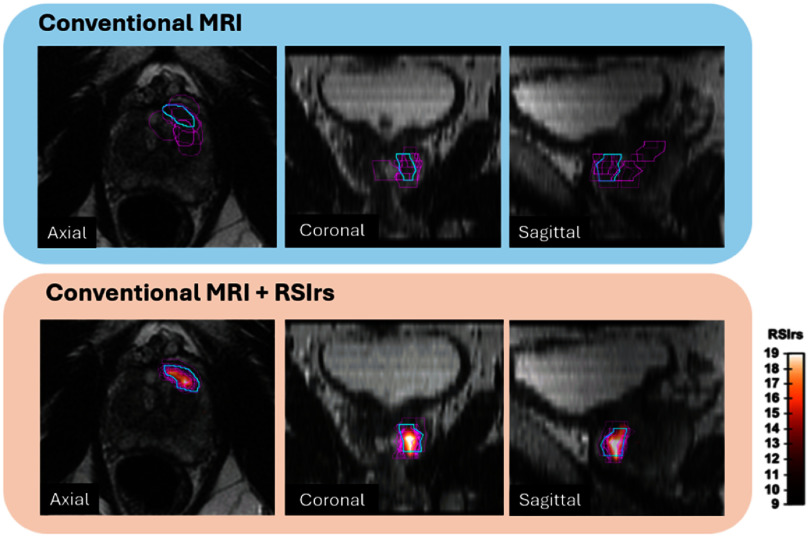
RSIrs maps improve the accuracy of participant target volumes. Expertly defined consensus volume is shown in cyan. Select participant volumes are highlighted in magenta. With RSIrs, participant volumes showed better agreement with the expert compared to conventional MRI alone.

ReIGNITE RT boost, a prospective international study, demonstrated that RSIrs maps help radiation oncologists more accurately identify focal boost target lesions when compared to expert-defined target lesions (Lui *et al* 2023). Without any training on interpretation of RSIrs maps, radiation oncologists were significantly more accurate when using RSIrs, with the rate of completely missed targets dropping from 19%–2%. Participants completely missed a median of 3 targets (out of 23) with conventional mpMRI, compared to 0 with RSIrs (figure 4). Furthermore, knowledge-based planning was used to demonstrate that using radiation oncologists’ contours yield a worse predicted probability of biochemical failure compared with expert-defined contours given high tumor contour variability. Addition of RSIrs decreased variability and improving accuracy (Zhong *et al* 2024). RSI acquisitions are fast (2–4 min) and do not require additional hardware or software to run on most 3 T clinical scanners marketed for diagnostic imaging, including baseline models from GE healthcare, siemens, and philips. RSI is already in use for diagnostic radiology at clinical imaging centers in the US and Europe, including academic and community settings. A version of RSI is FDA cleared and commercially available with the potential to impact patient care on a short timeline.

##### Current and future challenges

While there are still technical difficulties to consider when incorporating advanced diffusion strategies into a RT pipeline (such as anatomical distortions resulting from magnetic field inhomogeneities), these issues and the strategies used to overcome them have been thoroughly explored in prior sections of this manuscript. Instead, this portion will provide an overview of the difficulties that arise from the realities of clinical practice. Two major barriers to the use of DWI in RT include: (1) a lack of consensus as to what should be targeted on MRI for treatment and (2) a lack of adoption of prostate focal boost.

*Lack of consensus on how to target MRI-visible lesions*

To date, there is no consensus on how to contour prostate tumors, which is not a standard part of radiation oncology training. Evaluation of contours from both the FLAME trial as well as the ReIGNITE trial demonstrated the heterogeneity of radiation oncologist focal boost target delineation (van Schie *et al* 2018, Lui *et al* 2023, Zhong *et al* 2024). While use of RSIrs decreased this variability, there is a need for development of evidence-based guidelines for prostate tumor delineation on MRI.

*Lack of adoption of focal boost as a treatment strategy*

Recently, a global survey of radiation oncologists reported that overall, 43% of participants routinely used focal boost with 61% of complete genitourinary subspecialists reporting routine use (Zhong *et al* 2023b). Frequently cited barriers to implementation can be grouped into three categories: (1) concerns about relative clinical benefit versus toxicity, (2) concerns about MRI-CT registration/planning, and (3) challenges to access high-quality MRI.

##### Advances in science and technology to meet challenges

Recently, international consensus guidelines and a contouring atlas for MRI-delineation of the prostate bed have been developed with demonstrated improvement in contouring concordance (Sritharan *et al* 2024). Beyond only having consensus guidelines, atlases and education programs are needed to improve compliance to these guidelines, as seen in other tumor sites (Bekelman *et al* 2009). A recent systemic review offered various approaches to address the barriers limiting adoption of focal tumor boosting (Dornisch *et al* 2024). One recommendation is simply to raise awareness of the practice and highlight clinical trials that investigate its clinical benefit (Syndikus *et al* 2020, De Cock *et al* 2023). Concerns about MRI-CT registration/planning can be met with advances in MRI-only prostate RT workflows, MRI-simulation, automatization of MRI to CT registration using DL networks, and knowledge-based planning (Ciardo *et al* 2017, Ray *et al* 2020). Dissemination of accurate quantitative MRI, such as RSIrs, without need for local radiologist or oncologist expertise could increase access to high-quality MRI across the US and beyond.

##### Concluding remark

While DWI is currently used in a semi-quantitative manner in diagnosis and local staging of prostate cancer as well as prostate RT, this requires significant radiologist and oncologist expertise, which is not always available. In contrast, advanced quantitative DWI, such as RSIrs, can help fill the need for reproducible, accessible, and standardized imaging necessary for optimal and equitable prostate RT. However, there are technical barriers to implementing advanced DWI methods and a broader lack of consensus on how to contour prostate lesions that have limited the adoption of focal tumor boosting at many treatment centers. Efforts are currently underway to assuage these barriers and improve patient access to focal boost treatment.

#### Concluding statement for chapter III

*Tyler M Seibert*^1,2,3,4^ *and Jill B De Vis*^5,6^

^1^ Department of Radiation Medicine and Applied Sciences, University of California San Diego Health, La Jolla, CA, United States of America

^2^ Department of Radiology, University of California San Diego Health, La Jolla, CA, United States of America

^3^ Department of Bioengineering, University of California San Diego Jacobs School of Engineering, La Jolla, CA, United States of America

^4^ Department of Urology, University of California San Diego Health, La Jolla, CA, United States of America

^5^ Department of Radiation Oncology, Harold C. Simmons Comprehensive Cancer Center, University of Texas Southwestern Medical Center, Dallas, TX, United States of America

^6^ Advanced Imaging Research Center, University of Texas Southwestern Medical Center, TX, United States of America

DWI is a promising imaging biomarker with the potential to predict tumor response to treatment, identify subregions suitable for radiation dose escalation, and serve as a critical tool in BIgART. However, most studies to date have been small-scale and have reported inconsistent results, largely due to variability in image acquisition protocols, calculation methods, and threshold definitions used to characterize DWI abnormalities. Furthermore, in anatomically complex regions, DWI data tends to be less robust, introducing additional confounding factors. To facilitate the clinical integration of DWI, future efforts should prioritize the development of improved acquisition methods to enhance measurement robustness across different anatomical sites. Standardization of DWI protocols across institutions, the development of machine learning–based fitting and segmentation algorithms, and the execution of multi-center studies to assess repeatability and biological relevance will also be essential. By addressing these challenges, DWI can evolve from a research tool used primarily in group studies to a clinically actionable imaging modality for personalized radiation oncology, ultimately leading to improved patient outcomes. Ongoing prospective studies will be highly informative (e.g. NCT04349501, NCT04992728, NCT06990542, NCT04700748, NCT02059889, NCT03151642, NCT03145077).

## Chapter IV. Quality assurance/control, and clinical trials of DWI in RT

### 11. Protocol standardization and QA/QC for DWI in RT

#### H Michael Gach^1,2^ and Taeho Kim^1^

^1^ Department of Radiation Oncology, Washington University in St. Louis, St. Louis, Missouri, United States of America

^2^ Departments of Radiology and Biomedical Engineering, Washington University in St. Louis, St. Louis, Missouri, United States of America

##### Status

MRI diffusion represents a quantitative imaging biomarker that should be reproducible and consistent regardless of the pulse sequence and MRI platform. Quality assurance requires suitable phantoms, MRI protocols, analysis tools, and performance benchmarks (Boss *et al* 2024).

*QA phantoms*

**Figure 5.**
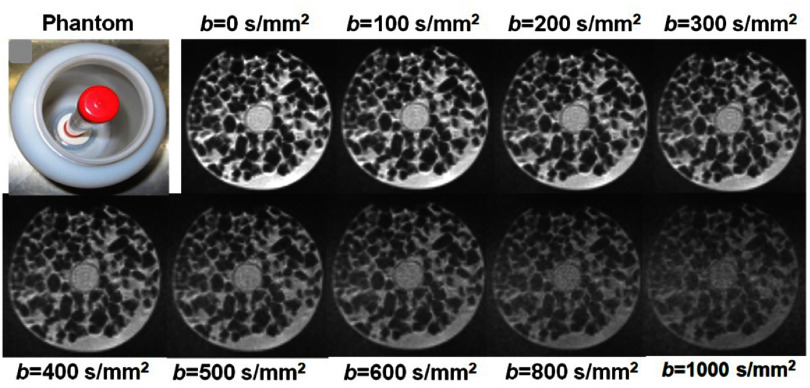
DWI images acquired in the American college of radiology imaging network (ACRIN) ice water phantom using the ViewRay 0.35 T MRI-^60^Co. The DWI images illustrate that the SNR drops as the *b*-value rises.

The American College of Radiology Imaging Network (ACRIN) was created in 1997 to organize imaging protocols for clinical trials that incorporated imaging biomarkers. ACRIN subsequently merged with the Eastern Cooperative Oncology Group (ECOG) to form ECOG-ACRIN, a member of the National Cancer Institute (NCI) national clinical trials network. Medical centers that participated in ACRIN clinical trials typically required site approvals using QA phantoms to verify satisfactory MRI performance. One of the original phantoms used in ACRIN diffusion MRI clinical trials (e.g. NRG BN-001) consisted of a 225 mm long, 27 mm diameter test tube containing distilled water in a 145 mm diameter ice bath (figure 5).

Ideally, a diffusion phantom should cover the range of ADC values seen *in vivo* from dense highly cellular tumors (e.g. 0.6 × 10^−3^ mm^2^ s^−1^) to cerebrospinal fluid (3.5 × 10^−3^ mm^2^ s^−1^) (Jafar *et al* 2016). The National Institute For Standards and Technology (NIST), the quantitative imaging biomarker alliance (QIBA), and the Radiological Society of North America (RSNA) developed a 194 mm diameter spherical shell diffusion phantom (CaliberMRI, Boulder, CO) with the aim of standardization and translation across MRI systems.

The ACRIN and QIBA phantoms are examples of phantoms created with institutional consensus and available for purchase from commercial sources. Other commercial sources of DWI and diffusion tensor imaging (DTI) phantoms include psychology software tools and HQ Imaging. However, a variety of homemade or locally made diffusion phantoms were created and used in diffusion QA studies. Compositions include polyvinylpyrrolidone (PVP), Taxon^TM^, polyacrylamide, and agarose. Regardless of the phantom used in diffusion MRI QA, the characterization, range, and stability of the diffusivity of the materials used in the phantom is critical (Boursianis *et al* 2021). The QIBA diffusion phantom contains ten 30 ml vials of aqueous PVP 40 kilo-Dalton (K40), and three 30 ml and three 5 ml vials of highly purified (high performance liquid chromatography) water. The original version of the phantom used ice water to maintain the samples at 0 °C. The latest version of the phantom includes a liquid crystal thermometer with a range of 17 °C–23 °C in increments of 1 °C to allow room temperature measurements. The diffusivity of water at 20 °C is 2.023 × 10^−3^ mm^2^ s^−1^ (Holz *et al* 2000). The thermometer can be read directly from the MRI images. A study of aqueous PVP K40 showed linear relationships between temperature and diffusivity and between concentration and diffusivity (Amouzandeh *et al* 2022). The manufacturer recommends replacing the diffusion phantom after three years in case the PVP dehydrates. ADCs were also measured on a ViewRay 0.35 T MR-LINAC using a diffusion phantom with 20 °C calibrated ADC values of 0.40, 1.00, 1.60, and 2.02 mm^2^ s^−1^ (HG Imaging, Heidelberg) (Wallimann *et al* 2024). QA phantoms are also available for measuring DTI, diffusion basis spectrum imaging, and Q-ball.

Protocol standardization is addressed by consensus statements (Padhani *et al* 2009, Baltzer *et al* 2020, Ljimani *et al* 2020, Dall’Armellina *et al* 2024). Recommendations are often specific to the target organ or disease. According to PubMed, the number of citations from 2007 through 2024 was 4123 for DWI in RT and 66 for consensus statements regarding DWI in RT. QIBA asserts that mean ADC variations exceeding the repeatability coefficient threshold (e.g. 8% for brain lesions, 27% for liver and prostate lesions, and 15% for breast lesions) are clinically significant with 95% confidence (Boss *et al* 2024).

##### Current and future challenges

The main priority and challenge for DWI is to ensure the measured ADCs are accurate and consistent within, and between, MRI systems. ADC reproducibility varies between MRIs based on vendor-specific sequences and parameters, SNR, RF coils, body habitus, and ADC computation. The accuracy of the diffusion encoding depends on the gradient linearity and eddy current compensation (Reese *et al* 2003, Bodammer *et al* 2004, Tao *et al* 2018). In principle, ADCs do not vary with magnetic field strength. Eddy current compensation typically includes both pre-emphasis (pulse shaping) to ensure gradient pulse fidelity, and image reconstruction techniques to correct quantitative errors associated with long-time constant eddy currents (Horsfield 1999, Zeilinger *et al* 2017, Valsamis *et al* 2022).

Philips uses a single-refocused spin echo while Siemens and GE use a TRSE to minimize the effects of eddy currents generated from the diffusion encoding gradients. However, the TRSE results in a longer ET, resulting in decreased SNR. Hence, an eddy current nulled convex optimized diffusion encoding using a single-refocused spin echo was demonstrated to provide similar DWI quality as the TRSE but with a short TE (Aliotta *et al* 2018). Pre-excitation gradients were also employed.

**Figure 6.**
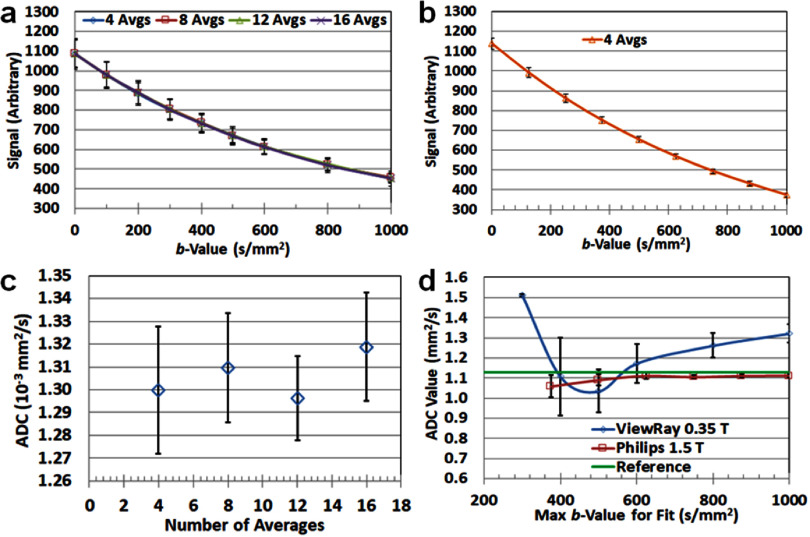
Results from American college of radiology imaging network (ACRIN) diffusion phantom for ViewRay 0.35 T MRI-^60^Co (running IDEA ICE VB19) and philips 1.5 T ingenia MRI (version 5.1.7). (a) ViewRay DWI signals for various averages versus *b*-value. (b) Philips DWI signal for four averages versus *b*-value. (c) ADC values for ViewRay for different signal averages fit using all *b*-values. (d) ADC values calculated using different maximum *b*-values. The reference line represents the correct ADC value for water at 0 °C. Error bars in (a) and (b) represent the standard deviations of the averages. Error bars in (c) and (d) represent the uncertainty of the fit.

Low SNR may be caused by the use of high *b*-values or low magnetic fields (figure 6). In principle, measured ADC values will be underestimated when SNR is low. The underestimation can be reduced by subtracting the Rician noise. The vendors typically acquire more repetitions (signal averages) for high *b*-values. The ACRIN phantom was used to compare ADC values measured on a Philips 1.5 T Ingenia and a ViewRay 0.35 T MRI-^60^Cobalt (figure 6). In the case of the ViewRay, diffusion encoding was along the slice direction due to limits in gradient amplitudes (a total of 17.99 mT m^−1^ regardless of the number of axes turned on) thus restricting *b*-values. ADC errors have also been addressed using MRI acceleration and denoising.

Every MRI system has significant gradient nonlinearities that result in spatial bias that rises with distance from isocenter. Geometric distortion scales proportionally with gradient nonlinearities. However, ADC errors rise quadratically with increasing gradient nonlinearities. Like geometric distortion, spatial ADC nonuniformities can be corrected based on quantification of the gradient nonlinearities. Post-acquisition corrections are often applied to correct gradient nonlinearities and residual (e.g. long) eddy currents from imperfect pre-emphasis. *B*_0_ eddy currents affect ADC values regardless of their spatial location. Gradient eddy currents and cross-term eddy currents can result in spatially variable effects on image distortion and ADC values.

The reference *b*= 0 s mm^−2^ acquisition may be replaced by a low-valued *b*-value (e.g. <1 s mm^−2^) so that crusher gradients can be used to dephase stimulated echoes. Higher reference *b* values (*b*= 50–100 s mm^−2^) are used to dephase bright (free diffusion) regions with high *T*_2_ signal (*T*_2_ shine-through).

**Figure 7.**
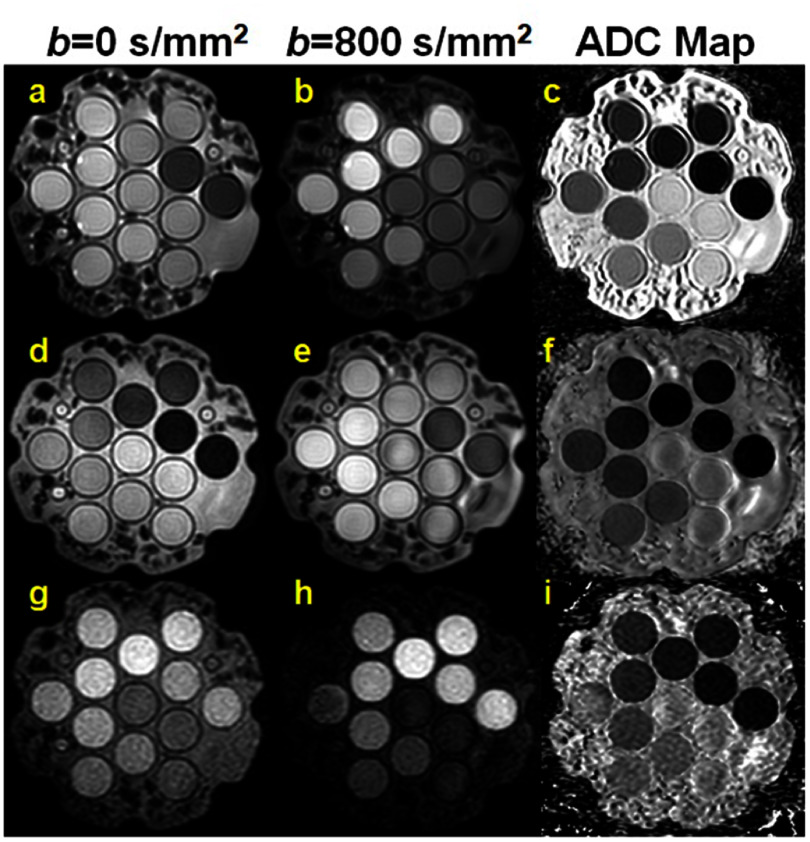
Quantitative imaging biomarker alliance (QIBA) phantom images from echo planar imaging (DWI-EPI, (a)–(c)), single-shot turbo spin echo (DWI-ssTSE, (d)–(f)), and split acquisition of fast spin-echo signals for diffusion imaging (DWI-SPLICE, (g)–(i) measured on a philips 1.5 T ingenia (software version 5.3.1). Left column (a), (d) and (g): *b* = 0 s mm^−2^, Center column (b), (e) and (h): *b* = 800 s mm^−2^, Right column (c), (f) and (i): *b* = 800 s mm^−2^.

Geometric distortion and chemical shift artifacts are typical of EPI acquisitions (figures 7(a)–(c)). Field maps can be used to reduce distortion. Acquisition with the readout gradient reversed also helped to reduce the distortion. Split acquisition of fast spin-echo signals for diffusion imaging (SPLICE) is a stimulated-echo technique that reportedly improves the fidelity of ADC values (figures 7(g)–(i)). However, the technique has reduced SNR compared to conventional single-shot TSE (DWI-ssTSE, figures 7(d)–(f)). In addition, the resulting ADC values may still vary from the standards. Recent enhancements to SPLICE have reportedly improved SNR and ADC accuracy (Rahbek *et al* 2023).

**Figure 8.**
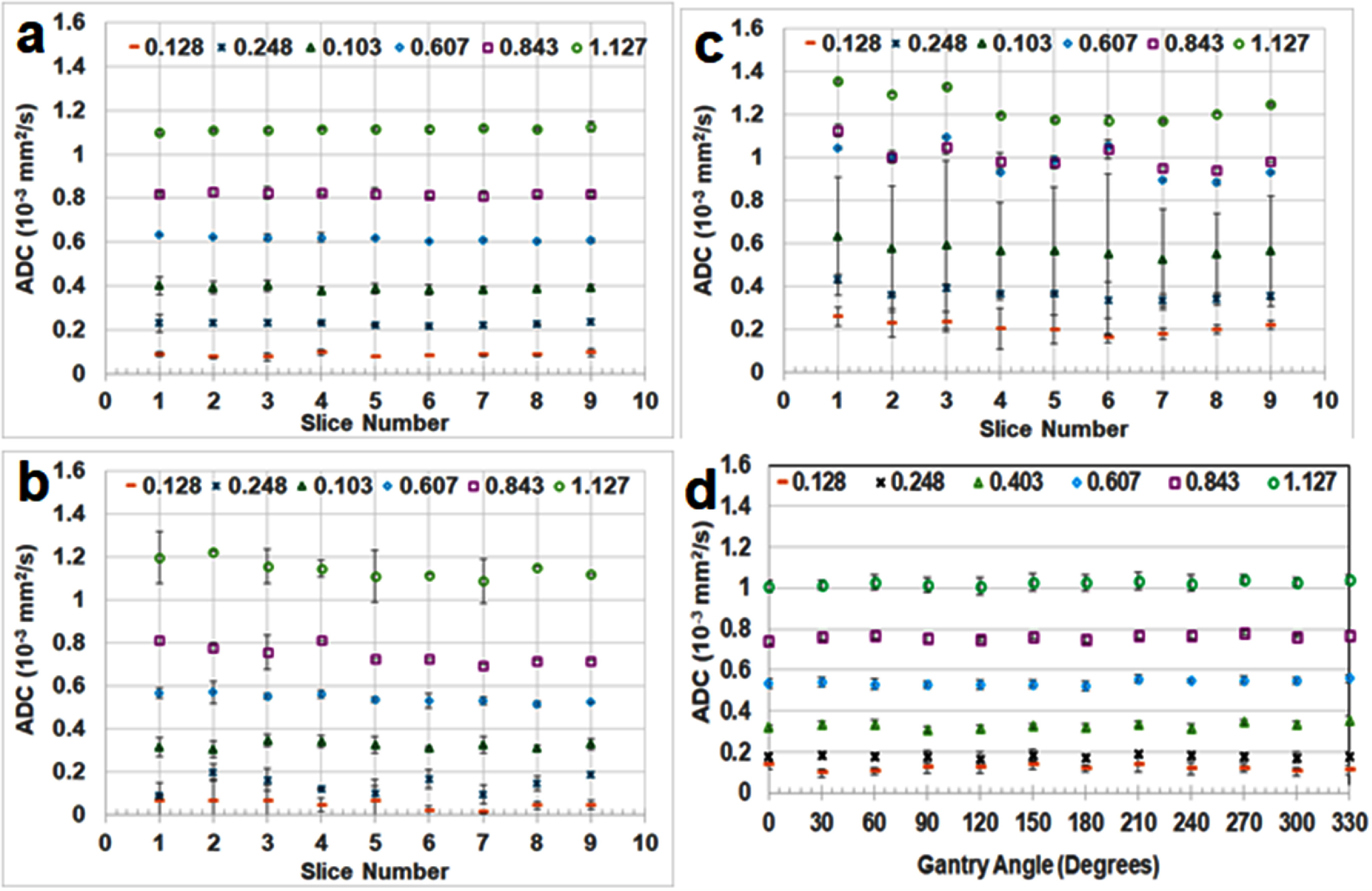
Trace ADCs measured in the NIST/QIBA diffusion phantom using (a) DWI-EPI, (b) DWI-ssTSE, and (c) DWI-SPLICE on a Philips 1.5 T Ingenia (software version 5.3.1). (d) Trace TRSE DWI-EPI ADC values versus gantry angle measured on a ViewRay 0.35 T MR-LINAC running IDEA/ICE VB19 (Lewis *et al* 2021). Error bars represent standard deviations. Adapted from Lewis *et al* (2021). CC BY 4.0.

MRI systems are often optimized for DWI-EPI. ADCs measured in the QIBA phantom using DWI-EPI were accurate and consistent across image slices while the DWI-ssTSE and DWI-SPLICE were not (figures 8(a)–(c)). TSE and multiband sequences are vulnerable to excitation crosstalk (Olson *et al* 2019). Use of slice gaps (10%–20% of slice thickness) or enhanced RF pulses reduce the ADC interslice variability (Pang *et al* 2022).

Fat saturation or suppression method may also contribute to variance in ADC measurements. Such techniques include chemical saturation, spectral presaturation with inversion recovery, spectral attenuated inversion recovery, and spectral spatial excitation pulses. The timing and interaction with image acquisition will vary for each.

*QA of DWI in MR-LINACs:* Multiple studies have examined the accuracy of ADCs measured on 0.35 T and 1.5 T MR-LINACs with varying results (Lawrence *et al* 2021, Lutsik *et al* 2024). In our study of the ViewRay MRIdian MR-LINAC, ADC values from TRSE DWI-EPI were typically underestimated and varied with gantry angle (figure 8(d)) (Lewis *et al* 2021). Significant gantry angle-dependent B_0_ eddy currents were measured on the ViewRay 0.35 T MR-LINAC (version 2) (Curcuru *et al* 2021). These eddy currents can cause isocenter shifts and perturb the desired *b*-value. Spatial variations in ADC values were also reported for the Elekta Unity 1.5 T MR-LINAC, suggesting gantry-related eddy currents (Kooreman *et al* 2020).

Consolidation of consensus statements for DWI in RT is needed to maximize the coverage of RT targets but minimize redundancy. Adoption of the consensus statements by RT societies like the american society for radiation oncology and european society for radiotherapy and oncology will enhance uniformity. The measured ADC values will depend on the chosen *b*-values, sequences, and diffusion models (e.g. Gaussian vs non-Gaussian) (Yablonskiy and Sukstanskii 2010, Baltzer *et al* 2020). In addition, region of interest (ROI) size and delineation decision-making affect quantification and standardization.

##### Advances in science and technology to meet challenges

Stronger gradients and improved compensation for gradient nonlinearities and eddy currents must be tailored for the system configuration. Bias correction for gradient non-linearities (or nonuniformities) were demonstrated to correct spatial variance in measured ADCs. Diffusion encoding on MR-LINACs is typically slower than diagnostic MRI systems since the MR-LINACs have lower gradient performance. On the Elekta Unity 1.5 T MRI, *b* values of ⩽500 s mm^−2^ were recommended to maintain a TE and achieve satisfactory ADC accuracies (Kooreman *et al* 2020).

Faster DWI sequences are required to shorten the patient MRI exam and minimize the effects of motion on artifacts and accuracy. Sparse acquisitions using compressed sensing or DL reconstructions can be expanded and evaluated using the QA tools.

Automated DWI analysis software tools are required to obtain rapid and reproducible results for phantoms and *in vivo* targets. Most of the current tools are optimized for phantoms (e.g. CaliberMRI’s qCal-MR®) or the brain (functional MRI of the brain software library) (Luna *et al* 2012). MRI vendor tools for diffusion data may not be adequate for quantitative diffusion measurements in the body. Such software tools must be reliable, robust, and accurate.

##### Concluding remarks

Diffusion phantoms serve an important role in verifying the performance of the DWI sequences and image processing for both MRI simulators and MR-LINACs. Phantom studies aid in quantifying the systemic sources of variance in diffusion measurements. However, *in vivo* diffusion measurements are subject to magnetic inhomogeneities, free, hindered, and restricted diffusion, and physiological motion. Future phantoms that incorporate these sources of variance can enhance the accuracy and utility of diffusion MRI as a quantitative imaging biomarker (Morozov *et al* 2020). Standardization of diffusion measurements *in vivo* benefits from consensus protocols and the lessons learned. However, the growing number of diffusion MRI protocols and targets encourages sites to make a choice in methods and to periodically assess its execution and clinical value.

### Acknowledgements

We acknowledge the support of National Institutes of Health (NIH) National Heart, Lung, and Blood Institute (NHLBI) Grant R01 HL148210.

### 12. Patient QA for DWI in radiation oncology practice

#### Joseph Weygand

Department of Radiation Oncology and Applied Sciences, Dartmouth College, Hanover NH, United States of America

##### Status

Ensuring the consistency and reliability of diffusion measurements is essential to achieving widespread utilization of DWI in clinical radiation oncology practice. While many publications note the importance of measurement stability, it is worth delving into the specific dimensions along which consistency is evaluated. First, the reproducibility of measurements is derived from the same scan when the ROI is delineated either by different clinicians or by the same clinician at different times. Here, the uncertainty stems primarily from intraobserver and interobserver variability rather than from true imaging differences (Shukla-Dave *et al* 2019).

Second, repeatability refers to the consistency of diffusion measurements acquired from the same patient or phantom on the same scanner under identical imaging conditions, either consecutively or across different days, assuming no true biological change has occurred (Miquel *et al* 2012, Spick *et al* 2016). Repeatability is principally limited by scanner hardware performance, including gradient stability, RF coil uniformity, and magnetic field homogeneity, although protocol parameters and operator technique can also contribute (NEMA 2008). It is important to note that variations in acquisition or reconstruction protocols are not considered within the scope of repeatability but fall under broader assessments of reproducibility.

Finally, inter-scanner reproducibility examines the consistency of measurements obtained across different scanners, where both hardware and software differences can substantially impact results (Grech-Sollars *et al* 2015). For DWI to serve as a quantitative imaging biomarker in clinical radiation oncology, each of these sources of variability must be rigorously assessed, minimized, and transparently reported.

Currently, the field has made progress in characterizing repeatability and reproducibility in conventional MRI platforms. Studies have reported within-subject coefficients of variation (wCV) for ADC values in the range of 3%–10% depending on the anatomical site and protocol standardization (Shukla-Dave *et al* 2019). However, the status of repeatability assessments on MR-LINACs remains limited. Early phantom studies suggested encouraging performance; for instance, a study conducted on a 0.35 T MR-LINAC demonstrated sub-5% standard deviation in ADC values across clinically relevant ranges (Weygand *et al* 2023). Yet, *in vivo* multi-institutional repeatability studies, particularly at the higher 1.5 T field strength, are largely lacking. Compounding this, geometric distortion, inherent to EPI-based DWI acquisitions, poses an elevated risk in radiation oncology applications where spatial accuracy is critical (Weygand *et al* 2016). Distortions not only misrepresent anatomy but can also lead to changes in ADC measurements, undermining clinical interpretation (Joint Head and Neck Radiotherapy-MRI Development Cooperative 2017). Thus, in addition to the phantom-based QA described previously, patient-specific monitoring for geometric fidelity and parameter adherence must be an integral part of DWI implementation in radiotherapy workflows.

##### Current and future challenges

Some of the current and future challenges hindering the widescale utilization of DWI in radiation oncology include the lack of standardization in ROI definition, insufficient repeatability and inter-scanner reproducibility studies on MRIgRT platforms, incomplete characterization of geometric distortions, and the limited availability of robust consensus protocols that are translatable across institutions. The segmentation of ROIs remains vulnerable to observer variation, although automatic segmentation tools are increasingly being developed to mitigate this. However, challenges exist not only in achieving scientific validation but also in navigating the regulatory pathways necessary for clinical deployment. Algorithms approved for specific scanners and acquisition parameters may not automatically generalize across different platforms without additional validation and regulatory approval (Muehlematter *et al* 2021).

In the realm of repeatability, while phantom studies offer foundational insight, broader *in vivo* investigations are necessary to truly establish the reliability of ADC as a clinical biomarker across the diverse hardware environments encountered in MRIgRT. Although no biological ground truth exists for ADC *in vivo*, such studies remain essential because they evaluate whether the biomarker exhibits stable and self-consistent behavior under the physiologic, anatomical, and workflow conditions unique to patient imaging, which cannot be reproduced in phantoms.

While efforts such as the Unity MR-LINAC consortium have begun to provide recommendations for managing distortion at 1.5 T (Kooreman *et al* 2020), comprehensive geometric distortion maps are still lacking for many clinical systems. Patient-specific assessment of geometric distortion introduces additional complexity beyond the system-level QA typically performed with phantoms. While CT–MRI comparisons can help identify gross spatial inconsistencies, their interpretation is constrained by physiologic motion, organ deformation, inherent uncertainties in fusion, and differences in patient setup between scans. MRI-based approaches such as B_0_-field mapping and reverse polarity gradient acquisitions offer more direct evaluation of susceptibility-related distortion. Anthropomorphic phantoms remain important for validating the overall imaging chain even if they are not used on a per-patient basis. As diffusion imaging becomes more widely incorporated into radiotherapy workflows, further development of structured and practical strategies for patient-specific distortion assessment will be necessary. Additionally, although most radiotherapy physicists are highly skilled in radiation physics, many do not possess advanced training in the subtleties of MRI physics and sequence optimization. As a result, sequence modifications and parameter optimizations often require collaboration with MRI physicists or reliance on well-vetted consensus protocols.

##### Advances in science and technology to meet challenges

Advances in science and technology are steadily addressing these challenges. The adoption of automatic segmentation tools, coupled with peer review and QA processes, offers a promising pathway to reduce observer-related variability. However, the full clinical translation of these tools demands careful validation and regulatory approval pathways, particularly in ensuring generalizability across scanners and imaging protocols. Progress is also being made in the standardization of diffusion imaging protocols through initiatives such as the QIBA, which seeks to harmonize acquisition parameters and reporting standards (Shukla-Dave *et al* 2019). Geometric distortion mitigation strategies, including the use of reduced FOV EPI, multi-shot techniques, and retrospective correction algorithms, are being increasingly explored and adapted to the unique constraints of MRIgRT platforms. It is essential that these technical advances be complemented by targeted educational efforts, fostering deeper collaboration between MRI and radiation oncology physicists to ensure protocol optimization is carried out thoughtfully and consistently. Optimized protocols must also be tailored to the anatomical site and adapted as necessary for individual patient body habitus, recognizing that a single protocol will not be universally appropriate across different clinical scenarios or scanner models.

##### Concluding remarks

The application of DWI into radiation oncology offers tremendous promise. The ability to interrogate tumor cellularity *in vivo* provides clinicians with insight into the physiology of a particular tumor. Biologically-guided radiotherapy treatments are achievable with DWI when the images are acquired in the treatment position, e.g. using MR-LINACs. However, a more rigorous characterization of the stability of the diffusion signal and the geometric fidelity of images are necessary for DWI to be implemented safely and effectively.

### 13. Clinical trial design incorporating DWI in RT

#### Yu-Feng Wang, Sirisha Tadimalla, Annette Haworth

Institute of Medical Physics, The University of Sydney, Sydney, Australia

##### Status

DWI is currently under investigation as a promising tool to improve RT across various cancers, including glioblastoma, HNC, and prostate cancer. Clinical trials are exploring the potential of DWI and its quantitative parameters as imaging biomarkers in two key applications: personalized RT planning and treatment response assessment during or after RT to determine the necessity for interventions such as treatment adaptation.

In the context of personalized RT planning, DWI is being investigated for its potential to provide imaging biomarkers of tumor location and biological characteristics. Although most studies remain in the investigational phase, some clinical trials have incorporated DWI into RT planning interventions. For example, the FLAME study utilized DWI in the RT planning phase to provide precise tumor location, thereby enabling a targeted focal boost of radiation (De Boer *et al* 2020). In non-interventional studies, quantitative parameters derived from DWI have demonstrated potential in personalizing RT by re-distributing radiation dose to escalate the dose in radioresistant tumor subregions (Zhao *et al* 2024) whilst sparing healthy tissue (Tadimalla *et al* 2022) to reduce treatment toxicity.

Another significant area of focus in current clinical trials is the use of DWI-derived quantitative parameters as imaging biomarkers for assessing treatment response, with the potential to inform adaptive RT. Several clinical studies have investigated the role of DWI in monitoring RT response across various cancers, including those in head and neck (El-Habashy *et al* 2023) and prostate (Wang *et al* 2023). These clinical trials are predominantly investigational, involving longitudinal imaging where DWI scans are acquired before treatment and at multiple time points during or after treatment. Typically, the statistical significance of changes in DWI-derived quantitative parameters, for example the ADC, is evaluated in comparison to baseline, pre-treatment changes. The primary objectives of these trials are to identify potential biomarkers of treatment response and, where sufficient data is available, to correlate changes in these quantitative parameters with clinical outcomes.

The design of existing clinical trials varies widely, presenting challenges in informing future studies to progress the use of DWI in RT. The MRI scanners used in these trials vary widely, including those from standard radiology departments, scanners dedicated to RT, and MR-LINAC systems. These studies are typically conducted either at a single institution or across multiple research institutions. Whilst the sequences employed are generally standard EPI-based sequences that are readily available on commercial scanners, the sequence implementation can differ between vendors. Furthermore, post-processing of the DWI varies between studies. Although commonly used parameters, like ADC, can often be calculated directly on the scanner, the underlying algorithm is often a black box, limiting transparency and reproducibility. On the other hand, parameters from more advanced models, like the IVIM model, are commonly calculated offline with inhouse developed software that is often not publicly available.

##### Current and future challenges

Designing future clinical trials that incorporate DWI in RT presents several challenges. A significant obstacle is the limited ability to fully utilize data from previous studies due to the lack of reported technical performance for the DWI-derived quantitative parameters, such as accuracy and precision (reproducibility and repeatability). Without this information, interpreting and comparing results across studies becomes challenging, impeding the ability to build on prior research effectively.

The reproducibility of quantitative parameters is influenced by several factors, including differences in the DWI sequences implementation across vendors, choice of DWI acquisition parameters (like *b*-value selection) and the specific post-processing steps employed. For example, a harmonization study on breast DWI showed that ADC values can vary up to 10% even when identical imaging protocols are implemented across scanners from different vendors, primarily due to system-dependent gradient nonlinearities (Malyarenko *et al* 2020). For other anatomical sites where organ motion or magnetic field inhomogeneity can be substantial, the variability can be much larger. Inaccuracies in *b*-value calibration arising from gradient nonlinearities (Posnansky *et al* 2011, Teh *et al* 2017, Lee *et al* 2020) and eddy current (Kooreman *et al* 2020), if not corrected, can directly propagate into errors in the estimated diffusion signal decay and consequently bias ADC measurements. Such effects become even more critical in advanced diffusion models that rely on multi-parameter fitting, where parameter estimates are inherently more sensitive to acquisition inaccuracies. Nevertheless, most clinical studies continue to rely on vendor-provided nominal *b*-values under the assumption of appropriate internal calibration. As a result, thresholds of DWI-derived quantitative parameter values may not be reproducible across different studies or even between scanners and institutions within the same study. This variability poses a significant challenge, as it hinders the ability to build on previous research findings and complicates the standardization necessary for widespread clinical application. While methods of protocol standardization and harmonization across scanners are actively being developed, further work is required to translate these into formal guidelines and practical tools for large-scale clinical trials.

Furthermore, not knowing the repeatability of these quantitative parameters poses a risk of overestimating their reliability as response biomarkers. For example, based on the results of a meta-analysis, the repeatability coefficient (%RC) of ADC in the prostate is 27%, (Boss *et al* 2024) which implies that changes within this range could be due to normal day-to-day variations in the scanner or the patient, rather than reflecting true treatment effects. Many studies report ADC changes during and after treatment that are comparable in magnitude to this estimation of the measurement uncertainty. Without assessing and reporting of the study-specific repeatability of the quantitative parameters, there remains insufficient evidence to support the technical robustness and feasibility of using them in larger, interventional clinical trials.

Another significant challenge is the limited evidence supporting the correlation between DWI-derived quantitative parameters and clinical outcomes, that is critical for validating these tools. Clinical outcome measures can require extensive collection of clinically meaningful outcomes and long-term follow-up data, particularly for cancers with longer prognosis times. Consequently, many existing studies rely on surrogate endpoints or are observational. Furthermore, practical issues, such as extended image acquisition times, can be burdensome for patients. Image quality concerns, particularly the susceptibility to distortion artefacts, also affect the confidence in deploying DWI-derived tools in interventional RT clinical trials. The complexity of DWI post-processing, often requiring bespoke software, poses additional hurdles when attempting to scale up to larger clinical trials or integrating these workflows into routine clinical practice.

##### Advances in science and technology to meet challenges

Advancing the utility of DWI in RT requires not only technological innovation but also consensus and collaboration within the research and clinical communities. While guidelines for assessing technical performance of DWI-derived quantitative parameters, such as those previously published by the QIBA (Shukla-Dave *et al* 2019), are available, they are often underutilized in clinical trial design. To acquire evidence on the reliability of DWI-derived quantitative parameters, it is crucial to establish a consensus on minimum standards for technical performance assessment and reporting in clinical trials.

For example, baseline assessment of accuracy and short-term repeatability in phantom objects should be conducted regardless of clinical trial design to provide a reference for future studies. Quality assurance programs, that have been demonstrated to assess reproducibility for multi-center trials (van Houdt *et al* 2020a) and to monitor the long-term stability of scanners in longitudinal trials (Wang *et al* 2021b), can also provide valuable reference to future clinical trial designs.

Furthermore, in clinical trials involving longitudinal DWI, *in vivo* test-retest imaging studies should be performed to validate the robustness of the measurements and the need to improve the measurement precision to detect potentially subtle treatment-related changes. Such test-retest studies do not necessarily need to be conducted in a separate cohort. For example, additional pre-treatment imaging sessions could be incorporated into the clinical trial design to obtain data for repeatability estimates in the same patient cohort.

Progression to the next stage of developing imaging biomarkers from DWI requires substantial clinical evidence provided through correlating imaging measurements with clinical outcomes. Conducting an independent clinical trial which involves collecting such data, particularly related to patient’s long-term outcomes, can be resource challenging. A viable alternative could involve integrating DWI as sub-studies within existing large-scale RT clinical trials, thereby leveraging established infrastructures for clinical data collection.

As imaging sequences and post-processing algorithms continue to evolve, DWI acquisition that utilizes more sophisticated models may be significantly accelerated, improving the clinical feasibility. Advancements in DWI technologies to improve the image quality are also likely to increase the reliability of the derived quantitative parameters, thereby growing the confidence in utilization of these tools in future interventional RT clinical trials. Consequently, rigorous testing and developing of the DWI protocol through phantom and *in vivo* imaging studies prior to the clinical trials are essential. The publication of such clinical trial datasets, with comprehensive imaging, clinical outcomes, and technical validation data, will play a crucial role in the development and validation of current and future imaging biomarkers derived from DWI for RT purposes.

##### Concluding remarks

The current era presents exciting opportunities as advances in imaging technologies such as DWI are being evaluated in clinical trials for integration into RT. Realizing this potential requires a rigorous approach to clinical trial design that includes technical validation. A collective commitment to technical performance assessment and reporting, ensuring consistency and standard across future clinical studies to advancing this field effectively. Collaborative efforts with larger interventional RT trials offer a promising avenue to gather DWI data alongside patient outcomes, which is crucial for the biological validation of these biomarkers. Consequently, the acquisition and validation process should be streamlined to enhance efficiency and facilitate broader adoption in clinical trials.

### Acknowledgements

This work was supported in part by the Westmead Charitable Trust and Cancer Institute NSW Early Career Fellowship Grant CINSW/2022/ECF1462. A Haworth is supported by a University of Sydney DVCR grant.

### Concluding statement for chapter IV

*H Michael Gach*

Department of Radiation Oncology, Washington University in St. Louis, St. Louis, Missouri, United States of America

Departments of Radiology and Biomedical Engineering, Washington University in St. Louis, St. Louis, Missouri, United States of America

DWI is currently a vital RT tool for both individual patient treatment management and clinical trials. The value of clinical DWI MRI for a wide range of RT applications resulted in a variety of approaches, each with its own set of quality benefits and limitations. A variety of phantoms are available to assess DWI quality before its integration in RT MRI protocols. Repeatability and reproducibility measurements must be performed to ensure the accuracy and efficacy of quantitative diffusion parameters. Providing quantitative information on inter-center discrepancies in diffusion parameter maps is essential. Such investigations require close collaboration among multiple centers to harmonize acquisition protocols and model-fitting methods, as well as dedicated test–retest studies—ideally conducted within the framework of prospective clinical trials or consortium-based initiatives. Clinical trials with DWI MRI require tackling the quality issues since equipment and pulse sequences will vary between vendors and clinical sites. Consensus statements can help simplify the choices of DWI technologies.

## Chapter V. Emerging innovations to enable biologically adaptive RT

### 14. Implementation of DWI on MR-LINAC

#### Petra J van Houdt

Department of Radiation Oncology, the Netherlands Cancer Institute, Amsterdam, the Netherlands

##### Status

Hybrid MR-LINAC systems allow frequent quantitative imaging without extra burden for the patient or costs (Hall *et al* 2019). This enables the use of DWI to adapt the treatment based on biological changes, i.e. BIgART. DWI is the most investigated technique so far on MR-LINAC systems (van Houdt *et al* 2024), with initial feasibility demonstrated on two commercial MR-LINAC systems (Hall *et al* 2019). This enables the use of DWI for BIgART (van Houdt *et al* 2021). DWI is the most investigated technique so far on MR-LINAC systems, with initial feasibility demonstrated on two commercial MR-LINAC systems (Kooreman *et al* 2019, Lewis *et al* 2021). The higher interest in DWI for BIgART compared to other quantitative MRI techniques is explained by the promising clinical evidence from studies on diagnostic systems showing that changes in ADC occur during treatment for many tumor sites (as discussed in sections 8–10) (van Houdt *et al* 2020b). It is important to note that DWI protocols from diagnostic scanners cannot simply be copied to MR-LINAC systems and need separate optimization to account for the reduced SNR and gradient strength/slew rate effects of the adapted MRI design (more details section 1) (Kooreman *et al* 2020, Nardini *et al* 2022).

As technical validation is a crucial aspect of biomarker validation (O’Connor *et al* 2017), demonstrating the performance of the DWI measurements on MR-LINAC systems is currently an active area of research. For a detailed summary of technical validation results we refer to a recent review paper (van Houdt *et al* 2024) and newer publications since then (Weygand *et al* 2023, Fernando *et al* 2024, Habrich *et al* 2024, Lutsik *et al* 2024, Rabe *et al* 2024, Wallimann *et al* 2024, Wong *et al* 2024). In general, good accuracy of ADC values is observed with diffusion phantoms at the iso-center of the MR-LINAC systems. However, larger ADC errors than on diagnostic systems are observed further away from iso-center. *In-vivo* repeatability of ADC values has been assessed in prostate, brain, and head-and-neck cancer (Lawrence *et al* 2021, Habrich *et al* 2022, McDonald *et al* 2023, Fernando *et al* 2024, Rabe *et al* 2024). The assessment of repeatability differs between studies, which makes the values difficult to compare. However, most studies report at least wCV or percent repeatability coefficient (%RC = 2.77 × wCV). Reported wCV values ranged between 0.9%–13.8% and reported %RC values range between 4.2%–31.3% (Lawrence *et al* 2021, McDonald *et al* 2023, Fernando *et al* 2024, Habrich *et al* 2024, Rabe *et al* 2024). In general, the intra-session repeatability is smaller compared to the inter-session repeatability (Lawrence *et al* 2021, McDonald *et al* 2023, Fernando *et al* 2024). Voxel-level repeatability coefficient was about 2–3 times as large as repeatability coefficient on region-level (mean ADC of a ROI) (Fernando *et al* 2024). For reference, %RC for a 1.5 T and 3 T diagnostic scanners was 13.3% in breast DWI (Newitt *et al* 2019). Differences between tumor sites might be ?> attributed to differences in protocol settings, the size of the region and motion that might be more apparent in e.g. the head and neck region compared to brain (Habrich *et al* 2022). A growing number of studies compared measurements on MR-LINAC systems to diagnostic systems with mixed results (Lawrence *et al* 2021, Habrich *et al* 2024, Lutsik *et al* 2024, Wallimann *et al* 2024, Wong *et al* 2024), where differences are mostly attributed to differences in protocol settings. Reproducibility can be improved with consensus about the *b*-values to be included in the analysis (Bisgaard *et al* 2023).

##### Current and future challenges

Sufficient repeatability of DWI parameters is crucial to be able to reliably measure changes during treatment. The repeatability coefficient should at least be lower than the expected effect size. For example, Lawrence *et al* showed that in 14 out of 20 glioblastoma patients treatment changes could be detected if that were larger than the repeatability coefficient (2021). Fernando *et al* showed that the voxel-level repeatability coefficient of ADC is much larger compared to repeatability coefficient of the mean ADC of an ROI (i.e. region-level) (2024). This makes it more difficult to use ADC for BIgART approaches where voxel-level interpretation of the ADC map is needed, for example boosting (i.e. higher dose) to radioresistant subvolumes of the tumor or for dose painting (i.e. dose level determined by the local ADC value). Several studies have shown that the repeatability is dependent on the size of the volume (Kooreman *et al* 2021, Habrich *et al* 2022), i.e. larger ROIs typically have a better repeatability coefficient, suggesting this is mostly an SNR issue. This is strengthened by the comparison of intra- and inter-fraction repeatability measurements in the prostate, showing that at voxel-level the repeatability is dominated by the intra-session effects rather than the inter-session effects such as misregistration and physiological variations (Fernando *et al* 2024). Therefore, efforts need to be directed towards improving SNR of DWI for MR-LINAC systems. This is especially important if more advanced DWI approaches are considered such as IVIM imaging (section 5) or restricted spectrum imaging (RSI; section 4).

In a typical clinical workflow, pre- and post-treatment imaging will be done on regular diagnostic systems. Therefore, the reproducibility of DWI parameters between MR-LINAC systems and diagnostic systems needs to be improved by harmonizing acquisition and analysis protocols between systems as well as institutes.

Tumors in the abdomen and thorax that move during breathing are a particularly relevant target for treatment on MR-LINAC systems as this allows real-time visualization of tumor position (Corradini *et al* 2019, Randall *et al* 2022). However, DWI in moving tissue is challenging due to respiratory as well as cardiac motion. Initial efforts towards implementation of DWI for moving targets are ongoing (Baas *et al*); however, more advanced acquisitions with motion compensated diffusion gradients might be needed (Starekova *et al* 2024). However, DWI in moving tissue is challenging due to respiratory as well as cardiac motion. Initial efforts towards implementation of DWI for moving targets are ongoing (Baas *et al*); however, more advanced acquisitions with motion compensated diffusion gradients might be needed.

Another challenge is increasing the acquisition speed. Currently, DWI data is typically acquired in the MR-idle time, during the time that is needed for plan adaptation to the daily situation. However, with clinical adoption of auto-contouring and planning, the overall treatment times will shorten thereby decreasing the opportunity time for quantitative imaging (Goodburn *et al* 2022). Therefore, faster acquisitions will be needed in the future.

##### Advances in science and technology to meet challenges

Improvement of SNR could be achieved via hardware or software improvements. For hardware improvements, more dedicated receive coils will improve the SNR (more details, see section 1) (Zijlema *et al* 2020). To make these available in clinical practice, development and implementation by the vendors is needed. For software improvements, DL is an obvious candidate to improve the SNR in DWI reconstructions (more details, see section 2) (Goodburn *et al* 2022). Commercial solutions are becoming available on diagnostic systems. A reduced bias and random measurement error of ADC values was obtained with DL-based reconstruction and denoising of low SNR DWI data (Lemainque *et al* 2024). However, these solutions are not yet available on MR-LINAC systems. Next to using DL solutions to improve image reconstruction, DL is also used for the quantitative analysis of DWI data and has the potential to improve the repeatability of the parameter maps (more details, see section 3) (Kaandorp *et al* 2021).

Vendor-neutral sequences are emerging as a research tool which allow using the same sequence design for different vendors (Karakuzu *et al* 2022). In this way, differences in sequence implementations by vendors are circumvented. For several quantitative MRI techniques, such as *T*_1_ mapping and magnetization transfer measurements, it has been shown that multicenter reproducibility improves when using vendor-neutral implementations compared to standard implementations (Karakuzu *et al* 2022). Similarly, the multivendor reproducibility was better for diffusion tensor parameters derived from vendor-neutral implementations of DWI for the brain (Liu *et al* 2024). Even though vendor-neutral sequences are currently a research solution, it might improve the multi-vendor reproducibility between MR-LINAC systems and diagnostic systems. More general, open science practices where code and data are shared, will help us to understand where differences between centers originate from and ultimately lead to reduced variability. A first step towards this goal is made with the IVIM code repository from the ISMRM OSIPI (https://github.com/OSIPI/TF2.4_IVIM-MRI_CodeCollection).

##### Concluding remarks

In conclusion, DWI acquisition is feasible on current clinical MR-LINAC systems. Due to adjusted MRI hardware of these systems as compared with diagnostic systems, dedicated DWI acquisition protocols have been developed. The performance of these measurements is currently being tested for several tumor sites. So far, diffusion phantom measurements have shown that the bias of ADC measurements is small close to the iso-center of the MR and comparable to diagnostic systems. The measured *in-vivo* repeatability varies between studies and tumor sites but is in general better for large regions compared to smaller regions. The repeatability at voxel-level seems insufficient to reliably detect changes, which seems mostly an SNR issue. To improve multi-center and multi-system reproducibility, harmonization of acquisition protocols and analysis procedures is needed.

### Acknowledgements

We would like to thank Prof. dr Uulke van der Heide for fruitful discussions on this topic.

### 15. The role of DWI in BIgART

#### Daniela Thorwarth

Section for Biomedical Physics, Department of Radiation Oncology, University of Tübingen, Tübingen, Germany

German Cancer Consortium, Partner site Tübingen, German Cancer Research Center, Heidelberg, Germany

##### Status

As DWI has been identified as candidate biomarker for BIgART, clinical research and translation is required to enable broad clinical use of this biomarker in future radiation oncology applications. Previous roadmaps have identified two major translational gaps associated with implementation and roll-out of imaging biomarkers in large multi-center trials or clinical practice which were associated with technical and clinical validation to ensure reliable measurement and with the availability of a biomarker for large-scale routine clinical usage (O’Connor *et al* 2017, van Houdt *et al* 2021). Recent review articles summarized multiple investigations regarding the technical and clinical validation of DWI on diagnostic but more importantly on combined MR-LINAC systems which are currently still ongoing (van Houdt *et al* 2020b, 2024). In addition, clinical availability and scalability of DWI as potential biomarker seems granted due to its availability for multiple field strengths and scanner types and the fact that no contrast-agent is required. Consequently, DWI seems to have the potential to successfully pass those two translational gaps, thus qualify as basis for testing the paradigm of BIgART in larger clinical trials. However, to date no prospective multi-center trials were carried out to investigate the potential of DWI-based BIgART for personalization of cancer radiotherapy.

##### Current and future challenges

For the clinical implementation of BIgART based on DWI, several prerequisites have to be fulfilled. First, DWI as a biomarker has to be linked to tumor biology. So far, only few studies were able to provide clear links between tumor biology and the measured DWI signal (Hompland *et al* 2018, Boeke *et al* 2023). Secondly, the biomarker information must be measured as accurately and repeatable as possible (section 14). Finally, a high reliability of the biomarker information must be given in terms of sensitivity and specificity. To date, several small single-center studies presented data regarding the prognostic value of DWI before, during or after RT (Wang *et al* 2021a, Martens *et al* 2022). Current studies often focus on the determination of an optimal time point for assessing RT response using DWI, also as basis for future BIgART interventions. With the availability of MR-LINAC where sequential DWI measurements during the course of treatment can be performed in parallel to RT application, several studies assessing DWI longitudinally were presented (Thorwarth and Muren 2019, Almansour *et al* 2023, Lawrence *et al* 2023, Bisgaard *et al* 2024). Lawrence *et al* (2023) found that volume changes of regions with low ADC inside the gross tumor volume (GTV) assessed between weeks two and five of RT were prognostic for overall survival and progression-free survival. The authors thus hypothesized that such low-ADC regions might qualify for dose painting BIgART. Similarly, Bisgaard *et al* (2024) demonstrated that both baseline and longitudinal DWI assessment were prognostic biomarkers for SBRT of pancreas carcinoma.

Only very few clinical studies testing the hypothesis of BIgART in patients were performed to date, consequently only little evidence on the predictive value of DWI-based BIgART exists. Fu *et al* (2022) reported results from a randomized, controlled clinical trial where 260 patients with locally advanced nasopharyngeal carcinoma were included to investigate the efficacy and toxicity of DWI-guided dose painting RT. In this trial, a subvolume defined by baseline ADC-values below the mean value was prescribed with a higher radiation dose. The study demonstrated that DWI-based dose painting RT was associated with significantly higher disease-free survival without increasing treatment-related toxicities (Fu *et al* 2022). A similar trial was reported by Mierzwa *et al* (2022), where BIgART was based on a combination of low blood flow assessed from DCE MRI and low ADC from DWI measured at baseline and in week two of RT. In this study, no improvement of overall survival following a physiology MRI-based BIgART boost could be shown, whereas a significant decrease in loco-regional failure rates was observed. These first BIgART studies demonstrated the feasibility of such concept and beneficial outcomes but did not investigate if a dose escalation to a specific biological volume was superior to applying the same boost to any other tumor region.

##### Advances in science and technology to meet challenges

Recent research has focused on biomarker identification, as well as technical and clinical validation of the prognostic character. However, research on optimal BIgART strategies is needed in order to investigate effective intervention strategies. So far, BIgART methodologies based on the definition of radioresistant subvolumes defined before or early during RT were investigated (Fu *et al* 2022, Mierzwa *et al* 2022, Boeke *et al* 2023). Mathematical models (more details, see section 16) may shed light on the efficacy of more complex DWI-based BIgART interventions in the future (Slavkova *et al* 2023). Novel concepts, such as optimal stopping in RT (OSRT) (Ajdari *et al* 2019, Ten Eikelder *et al* 2020) or spatiotemporal fractional RT (Torelli *et al* 2023) may be powerful approaches towards DWI-based BIgART in the future. OSRT approaches may also benefit from longitudinal continuous DWI measurements instead of discrete assessments in an offline setting. In addition, longitudinal correlation analyses to other functional assays may be informative to conceptualize future BIgART approaches (Wurschi *et al* 2023).

A currently open question is how to translate findings from patient cohorts, such as optimal imaging time points, imaging uncertainties or repeatability values to individual patient decisions. There might be quite high inter-patient variations, due to e.g. different pre-treatment ADC levels, differences in overall tumor volume or other physiological differences which need to be considered during DWI biomarker analysis and RT individualization. A study by Adjari *et al* (2022) presented a method for personalized BIgART treatment planning based on patient individual characteristics assessed via positron emission tomography by combining machine learning and optimization. Similar methodology may also be applicable to longitudinal DWI.

So far technical validation steps were only performed to interpret imaging data regarding single measurement time points. It remains unclear how this may translate to time series data. Potentially, published repeatability values may help to estimate optimal BIgART intervention timing or meaningful imaging time points during RT if no hybrid MR-LINAC is available and frequent longitudinal DWI assessment not possible. Furthermore, the impact of uncertainties originating from limited repeatability, accuracy and reproducibility on BIgART strategies are unclear and need to be investigated in future studies.

##### Concluding remarks

While technical validation of DWI parameters is ongoing, a major remaining challenge is associated with the second translational gap, which requires broad clinical availability and usability of DWI parameters as a biomarker. To achieve this, larger multi-center trials are required in order to prove that DWI can be used in a clinical context for BIgART. Furthermore, novel individualization concepts and BIgART approaches have to be investigated, especially to clarify the effect of dose escalation to biological sub-volumes in contrast to radiation dose effects only. To do so, open data, such as the recently published dataset of longitudinal DWI in head-and-neck cancer patients treated at the combined MR-LINAC (El-Habashy *et al* 2024), are essential to allow research on BIgART in many institutions around the world and benchmark mathematical models and ideas on common ground.

### 16. Using DWI for predictive mathematical modeling of tumor response to radiotherapy

#### David A Hormuth II^1,5^, Heiko Enderling^7^, Caroline Chung^7^ and Thomas E Yankeelov^1,2,3,4,5,6^

^1^ Oden Institute for Computational Engineering and Sciences, The University of Texas at Austin, Austin, Texas

^2^ Departments of Biomedical Engineering, The University of Texas at Austin, Austin, TX, United States of America

^3^ Diagnostic Medicine, The University of Texas at Austin, Austin, TX, United States of America

^4^ Oncology, The University of Texas at Austin, Austin, TX, United States of America

^5^ Livestrong Cancer Institutes, The University of Texas at Austin, Austin, TX, United States of America

^6^ Department of Imaging Physics, MD Anderson Cancer Center, Houston, TX, United States of America

^7^ Department of Radiation Oncology, MD Anderson Cancer Center, Houston, TX, United States of America

##### Status

The linear quadratic model is used clinically to compare the efficacies of different radiotherapeutic strategies, determine tumor control probability, and evaluate the therapeutic ratio between diseased and healthy appearing tissue (McMahon 2018). While this approach is utilized ubiquitously, the linear quadratic model is a very simplified approach based on single-cell survival that lacks any mechanistic clinical tissue response, any temporal component and any ability to personalize response for individual patients. Equally entrenched within clinical radiation oncology is medical imaging which is central to treatment planning, delivery, adaptation, and response assessment. While medical imaging can provide a rich snapshot of the underlying tumor biology at a particular point in time, it is limited to *assessing* response rather than *predicting* response.

To overcome these limitations, there has been much effort to link mathematical modeling with medical imaging to provide a mechanistic understanding of radiotherapy and therefore enable accurate, spatiotemporal predictions of the response to radiotherapy (Hormuth *et al* 2022). In particular, there have been attempts to address the shortcomings of the linear quadratic model and anatomical imaging through more biologically-complete mathematical descriptions of tumor cell response to radiotherapy (Enderling *et al* 2010, Hormuth *et al* 2022) parameterized by biologically-sensitive imaging techniques. Notably, DWI has been recognized as a promising tool in the field of predictive mathematical oncology, enabling non-invasive surrogate measures of tissue microstructure (e.g. white matter fiber tracks, diffusion anisotropy) (Painter and Hillen 2013) and cellular density (Hormuth *et al* 2021). As DWI is routinely collected as part of the clinical management of many malignancies, it offers the opportunity for personalization of mathematical models to individual patients to yield forecasts of not only treatment response but also optimizing the delivery of radiotherapy. Importantly, these methods are applicable to nearly all cancers that receive radiation.

##### Current and future challenges

With the advent of LINACs combined with MRI, treatment adaptation (see section 14) is also evolving towards daily ART based on superior soft tissue imaging and even biologically-informed adaptation based off DWI (see section 15) and other modalities (Bertholet *et al* 2021). Just as the introduction of LINACs combined with MRI (section 14) have enabled daily ART guided by biologically-informed imaging such as DWI (section 15) and other modalities (Bertholet *et al* 2021), the field of radiation oncology is poised for another transformational leap. This next step involves the integration of predictive mathematical modeling to personalize radiotherapy through an anticipatory approach, leveraging longitudinal biological imaging, including DWI, to optimize radiation treatment planning and improve patient outcomes (Enderling *et al* 2019). However, the promise of these mathematical modeling approaches has been demonstrated in highly controlled research settings where the imaging data is acquired consistently with quantitative metrology. Translation of these approaches to the broader clinical community will be challenged by the variability in imaging devices and protocols that will certainly impact the model predictions. For example, as longitudinal DWI forms the foundation of this modeling approach, it is essential that DWI is collected and analyzed in a fashion to yield repeatability and quantifiable DWI measures across different clinical sites. This challenge has been recognized by the RSNA’s QIBA and the NCI quantitative imaging network who have produced recommendations to imaging protocols and analyses to improve the precision and reliability of DWI (Shukla-Dave *et al* 2019). Similar challenges exist for other components of the mathematical modeling framework including registration and segmentation. This uncertainty and variability in imaging measures, imaging registration, and segmentation inevitably propagates through the predictive mathematical model and informs the confidence and trustworthiness of downstream predictions and decisions. To account for this uncertainty and enable our vision for predictive or model-based radiotherapy planning we have identified four major computational-clinical challenges that need to be addressed for successful translation of these predictive techniques to clinical practice:

(1) What data are necessary to be collected, how often, and when, to calibrate mathematical models for individual patients?
(2) How do we dynamically update model forecasts and treatment plans?
(3) What are the biologically-relevant targets for dose escalation or de-escalation?
(4) How do we present the outputs of the mathematical models to establish trustworthiness or confidence with clinical partners and patients?

##### Advances in science and technology to meet challenges

These four computational-clinical challenges can be reframed more broadly as challenges in experimental design, data assimilation, risk-informed decision making, and uncertainty quantification which are well-established tools across many engineering disciplines. These four entities are necessary components of a digital twin which is a virtual representation of a physical object that can be used to predict the behavior of the object and enable decision-making to optimize the future behavior of the object. Thus, a digital twin approach provides the mathematical and computational architecture to address these challenges and hasten the arrival of a new era of personalized radiation oncology. In oncology, we consider a digital twin a virtual representation of a patient’s tumor that captures bidirectional communication between the patient’s tumor in the physical and digital domains (National Academies of Sciences, Engineering, and Medicine *et al* 2024). This bidirectional communication consists of assimilating patient-specific data to initialize (and later dynamically update) the virtual representation (i.e. the computational domain, mathematical model parameters and variables) of the patient’s tumor and then returning risk-informed decisions on how to alter patient care (i.e. adapted radiotherapy plans, altered imaging schedule). This data assimilation component serves two purposes: first updating or personalizing the digital twin and providing guidance on when additional data (addressing challenge 1 via optimal experimental design (Cho *et al* 2020) may be needed given the data quality or the properties (e.g. growth rate) of the tumor itself and second dynamic updating of model forecasts given the current tumor state (addressing challenge 2). Underlying the bidirectional communication process is uncertainty quantification, which provides rigorous assessment of the uncertainty in the patient data, model parameters, and model predictions thereby enables trustworthiness in the safety and efficacy of a digital twin-proposed treatment adaptation. By integrating uncertainty quantification, the digital twin can offer dose escalation or de-escalation recommendations (challenge 3) with quantified confidence (challenge 4), supporting clinicians in making high stake-decisions.

**Figure 9.**
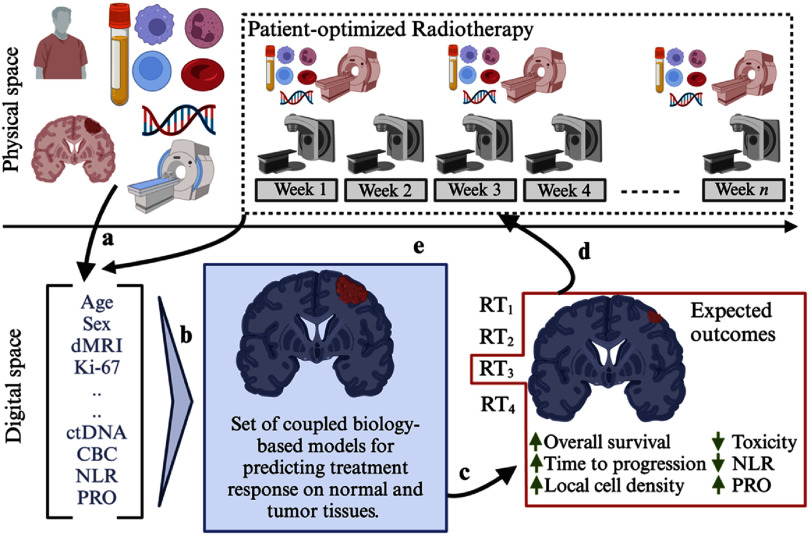
Visualization of a digital twin enabled framework for predicting and optimizing the response of patients to radiotherapy. Multi-modality patient imaging (DWI, *T*_2_ fluid attenuated inversion recovery, pre/post- contrast *T_1_*) and clinical data (Ki-67, ctDNA, CBC, NLR, PRO); arrow (a) are first used to define a digital representation of the patient and their tumor. This set of patient-specific features are used as inputs to a set of coupled biology-based models (arrow (b)) which characterize the dynamics of each feature, how each feature interacts with each other, and how it evolves in response to treatment. Patient-optimized radiotherapy regimens (RT_i_) can then be identified (arrow (c)) that yield the optimal efficacy and toxicity for an individual patient. For spatial-optimization of radiotherapy dose plans, tissue scale models driven by DWI report on local changes in cell density to identify under- (or over-) treated regions. This optimized radiotherapy regimen is then delivered to the patient in the physical space (arrow (d)). As more patient data (imaging or otherwise) is acquired throughout a patient’s care, the digital twin can be updated (arrow (e)), and the identification of optimal treatment regimens (arrows (b)–(d)) can be repeated to provided continued optimized care. Abbreviations: RT_i_ = radiotherapy protocol *i*, ctDNA = circulating DNA, CBC = complete blood cell count, NLR = neutrophil lymphocyte ratio, PRO = patient reported outcomes.

As this digital twin is informed by a patient’s own data and is dynamically updated throughout their care—it can account for variability in site-specific uncertainty in DWI and other components of the modeling framework. Figure 9 illustrates a digital twin of high-grade glioma applied to radiation oncology integrating multi-modality patient data with a DWI-based model of response. Integration of multi-modality patient data allows a more complete representation of the patient and their disease potentially enabling predictions of changes in 3D cell density (via a DWI-based model) and clinical quantities of interest such as patient reported outcomes, toxicity, and overall survival. As DWI is well studied in many malignancies, incorporated into predictive mathematical models, and has been employed for treatment adaptation, it is well suited to help establish patient specific digital twins for optimizing treatment outcomes.

The immense opportunities for digital twins in radiation oncology comes not only from their ability to simply forecast outcomes for individual patients, but also their ability to evaluate any number of radiotherapy optimizations of spatial dose and schedule, alone or in combination with targeted agents or immunotherapeutics, *in silico* to identify the optimization with the highest probability of (for example) improving overall survival, minimizing normal tissue complications, or maximizing anti-tumor immune response. As the digital twin is built on a mathematical description of the underlying biology and physics of tumor response to radiotherapy it is also possible to interpret why specific areas need to be targeted for dose escalation or why a proposed treatment optimization may fail. This interpretability is critical not only for scientific rigor, but also for establishing trustworthiness and confidence (challenge 4) among clinical partners and patients, as it allows digital twin recommendations (challenge 1 and 3) to be presented transparently, with clear biological rational and quantified confidence in prediction outcomes.

The successful implementation of such digital twins will also allow for novel *in silico* clinical trials on digital twin cohorts. Such virtual trials can simulate different therapies on the identical digital patient, multiple times to estimate the impact of intra- and inter-patient heterogeneity and identify what treatments work best for the total population, and what approaches are more promising for patients that are not average.

##### Concluding remarks

Technical and computational developments have now made it possible to integrate advanced DWI data into predictive mathematical models of tumor growth and response. Future development of a digital twin framework could account for intra- and inter-patient heterogeneity in tumor growth, treatment response, and normal tissue toxicities to deliver optimized radiotherapy regimens. This model-enabled approach—driven by a patient’s own data—is suitable for both aggressive tumors which require increased and targeted doses to achieve tumor control as well as tumors which could be controlled with reduced total doses. As nearly half of all malignancies receive radiotherapy, the potential impact on oncology is undeniable. However, the accuracy and robustness of digital twin modeling hinges on the fidelity of quantitative MRI data used to inform and constrain model parameterization. Continued advancement in DWI acquisition and analysis techniques to fully realize the clinical potential of biologically-informed digital twin models. Notably, a key strength of a digital twin framework lies in its ability to perform end-to-end uncertainty quantification—from multi-modality data to the mathematical model predictions—which may help mitigate the impact of variability in data quality or model assumptions. This integrative approach positions digital twins as a potentially powerful tool for enabling precise and ART.

#### Acknowledgements

This work was supported through funding from the National Cancer Institute via R01 CA235800, U24CA226110, U01CA174706, U01CA244100, U01CA280849, and R01CA260003, and the Cancer Prevention and Research Institute of Texas via RP220225. Thomas E. Yankeelov is a CPRIT Scholar in Cancer Research. Caroline Chung is supported by the Andrew Sabin Family Foundation Fellowship, MD Anderson Cancer Center Institutional Research Grant and CCSG Radiation Oncology and Cancer Imaging Program, and the Marnie Rose Foundation.

### Concluding statement for chapter V

*Petra J van Houdt*^1^ *and David A Hormuth II*^2,3^

^1^ Department of Radiation Oncology, the Netherlands Cancer Institute, Amsterdam, The Netherlands

^2^ Oden Institute for Computational Engineering and Sciences, The University of Texas at Austin, Austin, TX

^3^ Livestrong Cancer Institutes, The University of Texas at Austin, Austin, TX, United States of America

Together, these developments position DWI as a critical enabler of biologically-informed RT. By integrating DWI into adaptive workflows, BIgART has the potential to shift radiation oncology from reactive treatment adjustments to proactive, personalized interventions. Continued advances in imaging protocols, biophysical modeling, and validation across platforms are needed and will be essential for clinical adoption. As chapter V illustrates, DWI-guided BIgART and digital twin technology represents a promising and transformative direction for precision radiotherapy. However, advances in these technologies need to coordinate with imaging scientists, clinicians, physicists, engineers, data scientists, patients and patient advocates to realize their full potential and acceptance. With rigorous evaluation and thoughtful integration, BIgART could redefine how treatment is tailored to the dynamic biology of each patient’s tumor. While several efforts demonstrate encouraging early-state results, true clinical readiness remains dependent on multi-site validation and integration with clinical decision-making pipelines. Ultimately, prospective trials designed to demonstrate clinical utility at the individual level are needed to establish theses methodologies in clinical practice.

## Data Availability

No new data were created or analysed in this study.
